# Measurement of b hadron lifetimes in pp collisions at $$\sqrt{s} = 8$$$$\,\text {Te}\text {V}$$

**DOI:** 10.1140/epjc/s10052-018-5929-3

**Published:** 2018-06-07

**Authors:** A. M. Sirunyan, A. Tumasyan, W. Adam, F. Ambrogi, E. Asilar, T. Bergauer, J. Brandstetter, E. Brondolin, M. Dragicevic, J. Erö, M. Flechl, M. Friedl, R. Frühwirth, V. M. Ghete, J. Grossmann, J. Hrubec, M. Jeitler, A. König, N. Krammer, I. Krätschmer, D. Liko, T. Madlener, I. Mikulec, E. Pree, N. Rad, H. Rohringer, J. Schieck, R. Schöfbeck, M. Spanring, D. Spitzbart, W. Waltenberger, J. Wittmann, C.-E. Wulz, M. Zarucki, Y. Dydyshka, V. Mossolov, J. Suarez Gonzalez, E. A. De Wolf, D. Di Croce, X. Janssen, J. Lauwers, H. Van Haevermaet, P. Van Mechelen, N. Van Remortel, S. Abu Zeid, F. Blekman, J. D’Hondt, I. De Bruyn, J. De Clercq, K. Deroover, G. Flouris, D. Lontkovskyi, S. Lowette, S. Moortgat, L. Moreels, Q. Python, K. Skovpen, S. Tavernier, W. Van Doninck, P. Van Mulders, I. Van Parijs, D. Beghin, H. Brun, B. Clerbaux, G. De Lentdecker, H. Delannoy, B. Dorney, G. Fasanella, L. Favart, R. Goldouzian, A. Grebenyuk, G. Karapostoli, T. Lenzi, J. Luetic, T. Maerschalk, A. Marinov, A. Randle-conde, T. Seva, C. Vander Velde, P. Vanlaer, D. Vannerom, R. Yonamine, F. Zenoni, F. Zhang, A. Cimmino, T. Cornelis, D. Dobur, A. Fagot, M. Gul, I. Khvastunov, D. Poyraz, C. Roskas, S. Salva, M. Tytgat, W. Verbeke, N. Zaganidis, H. Bakhshiansohi, O. Bondu, S. Brochet, G. Bruno, C. Caputo, A. Caudron, P. David, S. De Visscher, C. Delaere, M. Delcourt, B. Francois, A. Giammanco, M. Komm, G. Krintiras, V. Lemaitre, A. Magitteri, A. Mertens, M. Musich, K. Piotrzkowski, L. Quertenmont, A. Saggio, M. Vidal Marono, S. Wertz, J. Zobec, N. Beliy, W. L. AldáJúnior, F. L. Alves, G. A. Alves, L. Brito, M. Correa Martins, C. Hensel, A. Moraes, M. E. Pol, P. Rebello Teles, E. Belchior Batista Das Chagas, W. Carvalho, J. Chinellato, E. Coelho, E. M. Da Costa, G. G. Da Silveira, D. De Jesus Damiao, S. Fonseca De Souza, L. M. Huertas Guativa, H. Malbouisson, M. Melo De Almeida, C. Mora Herrera, L. Mundim, H. Nogima, L. J. Sanchez Rosas, A. Santoro, A. Sznajder, M. Thiel, E. J. Tonelli Manganote, F. Torres Da Silva De Araujo, A. Vilela Pereira, S. Ahuja, C. A. Bernardes, T. R. Fernandez Perez Tomei, E. M. Gregores, P. G. Mercadante, S. F. Novaes, Sandra S. Padula, D. Romero Abad, J. C. Ruiz Vargas, A. Aleksandrov, R. Hadjiiska, P. Iaydjiev, M. Misheva, M. Rodozov, M. Shopova, G. Sultanov, A. Dimitrov, I. Glushkov, L. Litov, B. Pavlov, P. Petkov, W. Fang, X. Gao, L. Yuan, M. Ahmad, J. G. Bian, G. M. Chen, H. S. Chen, M. Chen, Y. Chen, C. H. Jiang, D. Leggat, H. Liao, Z. Liu, F. Romeo, S. M. Shaheen, A. Spiezia, J. Tao, C. Wang, Z. Wang, E. Yazgan, H. Zhang, S. Zhang, J. Zhao, Y. Ban, G. Chen, Q. Li, S. Liu, Y. Mao, S. J. Qian, D. Wang, Z. Xu, C. Avila, A. Cabrera, L. F. Chaparro Sierra, C. Florez, C. F. González Hernández, J. D. Ruiz Alvarez, B. Courbon, N. Godinovic, D. Lelas, I. Puljak, P. M. Ribeiro Cipriano, T. Sculac, Z. Antunovic, M. Kovac, V. Brigljevic, D. Ferencek, K. Kadija, B. Mesic, A. Starodumov, T. Susa, M. W. Ather, A. Attikis, G. Mavromanolakis, J. Mousa, C. Nicolaou, F. Ptochos, P. A. Razis, H. Rykaczewski, M. Finger, M. Finger, E. Carrera Jarrin, Y. Assran, S. Elgammal, A. Mahrous, R. K. Dewanjee, M. Kadastik, L. Perrini, M. Raidal, A. Tiko, C. Veelken, P. Eerola, H. Kirschenmann, J. Pekkanen, M. Voutilainen, T. Järvinen, V. Karimäki, R. Kinnunen, T. Lampén, K. Lassila-Perini, S. Lehti, T. Lindén, P. Luukka, E. Tuominen, J. Tuominiemi, J. Talvitie, T. Tuuva, M. Besancon, F. Couderc, M. Dejardin, D. Denegri, J. L. Faure, F. Ferri, S. Ganjour, S. Ghosh, A. Givernaud, P. Gras, G. Hamel de Monchenault, P. Jarry, I. Kucher, C. Leloup, E. Locci, M. Machet, J. Malcles, G. Negro, J. Rander, A. Rosowsky, M. Ö. Sahin, M. Titov, A. Abdulsalam, C. Amendola, I. Antropov, S. Baffioni, F. Beaudette, P. Busson, L. Cadamuro, C. Charlot, R. Granier de Cassagnac, M. Jo, S. Lisniak, A. Lobanov, J. Martin Blanco, M. Nguyen, C. Ochando, G. Ortona, P. Paganini, P. Pigard, R. Salerno, J. B. Sauvan, Y. Sirois, A. G. Stahl Leiton, T. Strebler, Y. Yilmaz, A. Zabi, A. Zghiche, J.-L. Agram, J. Andrea, D. Bloch, J.-M. Brom, M. Buttignol, E. C. Chabert, N. Chanon, C. Collard, E. Conte, X. Coubez, J.-C. Fontaine, D. Gelé, U. Goerlach, M. Jansová, A.-C. Le Bihan, N. Tonon, P. Van Hove, S. Gadrat, S. Beauceron, C. Bernet, G. Boudoul, R. Chierici, D. Contardo, P. Depasse, H. ElMamouni, J. Fay, L. Finco, S. Gascon, M. Gouzevitch, G. Grenier, B. Ille, F. Lagarde, I. B. Laktineh, M. Lethuillier, L. Mirabito, A. L. Pequegnot, S. Perries, A. Popov, V. Sordini, M. Vander Donckt, S. Viret, T. Toriashvili, I. Bagaturia, C. Autermann, L. Feld, M. K. Kiesel, K. Klein, M. Lipinski, M. Preuten, C. Schomakers, J. Schulz, T. Verlage, V. Zhukov, A. Albert, E. Dietz-Laursonn, D. Duchardt, M. Endres, M. Erdmann, S. Erdweg, T. Esch, R. Fischer, A. Güth, M. Hamer, T. Hebbeker, C. Heidemann, K. Hoepfner, S. Knutzen, M. Merschmeyer, A. Meyer, P. Millet, S. Mukherjee, T. Pook, M. Radziej, H. Reithler, M. Rieger, F. Scheuch, D. Teyssier, S. Thüer, G. Flügge, B. Kargoll, T. Kress, A. Künsken, J. Lingemann, T. Müller, A. Nehrkorn, A. Nowack, C. Pistone, O. Pooth, A. Stahl, M. Aldaya Martin, T. Arndt, C. Asawatangtrakuldee, K. Beernaert, O. Behnke, U. Behrens, A. Bermúdez Martínez, A. A. Bin Anuar, K. Borras, V. Botta, A. Campbell, P. Connor, C. Contreras-Campana, F. Costanza, C. Diez Pardos, G. Eckerlin, D. Eckstein, T. Eichhorn, E. Eren, E. Gallo, J. Garay Garcia, A. Geiser, A. Gizhko, J. M. Grados Luyando, A. Grohsjean, P. Gunnellini, M. Guthoff, A. Harb, J. Hauk, M. Hempel, H. Jung, A. Kalogeropoulos, M. Kasemann, J. Keaveney, C. Kleinwort, I. Korol, D. Krücker, W. Lange, A. Lelek, T. Lenz, J. Leonard, K. Lipka, W. Lohmann, R. Mankel, I.-A. Melzer-Pellmann, A. B. Meyer, G. Mittag, J. Mnich, A. Mussgiller, E. Ntomari, D. Pitzl, A. Raspereza, B. Roland, M. Savitskyi, P. Saxena, R. Shevchenko, S. Spannagel, N. Stefaniuk, G. P. Van Onsem, R. Walsh, Y. Wen, K. Wichmann, C. Wissing, O. Zenaiev, R. Aggleton, S. Bein, V. Blobel, M. Centis Vignali, T. Dreyer, E. Garutti, D. Gonzalez, J. Haller, A. Hinzmann, M. Hoffmann, A. Karavdina, R. Klanner, R. Kogler, N. Kovalchuk, S. Kurz, T. Lapsien, I. Marchesini, D. Marconi, M. Meyer, M. Niedziela, D. Nowatschin, F. Pantaleo, T. Peiffer, A. Perieanu, C. Scharf, P. Schleper, A. Schmidt, S. Schumann, J. Schwandt, J. Sonneveld, H. Stadie, G. Steinbrück, F. M. Stober, M. Stöver, H. Tholen, D. Troendle, E. Usai, L. Vanelderen, A. Vanhoefer, B. Vormwald, M. Akbiyik, C. Barth, S. Baur, E. Butz, R. Caspart, T. Chwalek, F. Colombo, W. De Boer, A. Dierlamm, B. Freund, R. Friese, M. Giffels, D. Haitz, F. Hartmann, S. M. Heindl, U. Husemann, F. Kassel, S. Kudella, H. Mildner, M. U. Mozer, Th. Müller, M. Plagge, G. Quast, K. Rabbertz, M. Schröder, I. Shvetsov, G. Sieber, H. J. Simonis, R. Ulrich, S. Wayand, M. Weber, T. Weiler, S. Williamson, C. Wöhrmann, R. Wolf, G. Anagnostou, G. Daskalakis, T. Geralis, V. A. Giakoumopoulou, A. Kyriakis, D. Loukas, I. Topsis-Giotis, G. Karathanasis, S. Kesisoglou, A. Panagiotou, N. Saoulidou, K. Kousouris, I. Evangelou, C. Foudas, P. Kokkas, S. Mallios, N. Manthos, I. Papadopoulos, E. Paradas, J. Strologas, F. A. Triantis, M. Csanad, N. Filipovic, G. Pasztor, O. Surányi, G. I. Veres, G. Bencze, C. Hajdu, D. Horvath, Á. Hunyadi, F. Sikler, V. Veszpremi, A. J. Zsigmond, N. Beni, S. Czellar, J. Karancsi, A. Makovec, J. Molnar, Z. Szillasi, M. Bartók, P. Raics, Z. L. Trocsanyi, B. Ujvari, S. Choudhury, J. R. Komaragiri, S. Bahinipati, S. Bhowmik, P. Mal, K. Mandal, A. Nayak, D. K. Sahoo, N. Sahoo, S. K. Swain, S. Bansal, S. B. Beri, V. Bhatnagar, R. Chawla, N. Dhingra, A. K. Kalsi, A. Kaur, M. Kaur, S. Kaur, R. Kumar, P. Kumari, A. Mehta, J. B. Singh, G. Walia, Ashok Kumar, Aashaq Shah, A. Bhardwaj, S. Chauhan, B. C. Choudhary, R. B. Garg, S. Keshri, A. Kumar, S. Malhotra, M. Naimuddin, K. Ranjan, R. Sharma, R. Bhardwaj, R. Bhattacharya, S. Bhattacharya, U. Bhawandeep, S. Dey, S. Dutt, S. Dutta, S. Ghosh, N. Majumdar, A. Modak, K. Mondal, S. Mukhopadhyay, S. Nandan, A. Purohit, A. Roy, D. Roy, S. Roy Chowdhury, S. Sarkar, M. Sharan, S. Thakur, P. K. Behera, R. Chudasama, D. Dutta, V. Jha, V. Kumar, A. K. Mohanty, P. K. Netrakanti, L. M. Pant, P. Shukla, A. Topkar, T. Aziz, S. Dugad, B. Mahakud, S. Mitra, G. B. Mohanty, N. Sur, B. Sutar, S. Banerjee, S. Bhattacharya, S. Chatterjee, P. Das, M. Guchait, Sa. Jain, S. Kumar, M. Maity, G. Majumder, K. Mazumdar, T. Sarkar, N. Wickramage, S. Chauhan, S. Dube, V. Hegde, A. Kapoor, K. Kothekar, S. Pandey, A. Rane, S. Sharma, S. Chenarani, E. Eskandari Tadavani, S. M. Etesami, M. Khakzad, M. Mohammadi Najafabadi, M. Naseri, S. Paktinat Mehdiabadi, F. Rezaei Hosseinabadi, B. Safarzadeh, M. Zeinali, M. Felcini, M. Grunewald, M. Abbrescia, C. Calabria, A. Colaleo, D. Creanza, L. Cristella, N. De Filippis, M. De Palma, F. Errico, L. Fiore, G. Iaselli, S. Lezki, G. Maggi, M. Maggi, G. Miniello, S. My, S. Nuzzo, A. Pompili, G. Pugliese, R. Radogna, A. Ranieri, G. Selvaggi, A. Sharma, L. Silvestris, R. Venditti, P. Verwilligen, G. Abbiendi, C. Battilana, D. Bonacorsi, L. Borgonovi, S. Braibant-Giacomelli, R. Campanini, P. Capiluppi, A. Castro, F. R. Cavallo, S. S. Chhibra, G. Codispoti, M. Cuffiani, G. M. Dallavalle, F. Fabbri, A. Fanfani, D. Fasanella, P. Giacomelli, C. Grandi, L. Guiducci, S. Marcellini, G. Masetti, A. Montanari, F. L. Navarria, A. Perrotta, A. M. Rossi, T. Rovelli, G. P. Siroli, N. Tosi, S. Albergo, S. Costa, A. Di Mattia, F. Giordano, R. Potenza, A. Tricomi, C. Tuve, G. Barbagli, K. Chatterjee, V. Ciulli, C. Civinini, R. D’Alessandro, E. Focardi, P. Lenzi, M. Meschini, S. Paoletti, L. Russo, G. Sguazzoni, D. Strom, L. Viliani, L. Benussi, S. Bianco, F. Fabbri, D. Piccolo, F. Primavera, V. Calvelli, F. Ferro, E. Robutti, S. Tosi, A. Benaglia, L. Brianza, F. Brivio, V. Ciriolo, M. E. Dinardo, P. Dini, S. Fiorendi, S. Gennai, A. Ghezzi, P. Govoni, M. Malberti, S. Malvezzi, R. A. Manzoni, D. Menasce, L. Moroni, M. Paganoni, K. Pauwels, D. Pedrini, S. Pigazzini, S. Ragazzi, N. Redaelli, T. Tabarelli de Fatis, S. Buontempo, N. Cavallo, S. Di Guida, F. Fabozzi, F. Fienga, A. O. M. Iorio, W. A. Khan, L. Lista, S. Meola, P. Paolucci, C. Sciacca, F. Thyssen, P. Azzi, L. Benato, D. Bisello, A. Boletti, R. Carlin, A. Carvalho Antunes De Oliveira, P. Checchia, M. Dall’Osso, P. De Castro Manzano, T. Dorigo, U. Dosselli, F. Gasparini, U. Gasparini, A. Gozzelino, S. Lacaprara, P. Lujan, M. Margoni, A. T. Meneguzzo, M. Passaseo, M. Pegoraro, N. Pozzobon, P. Ronchese, R. Rossin, F. Simonetto, M. Zanetti, G. Zumerle, A. Braghieri, A. Magnani, P. Montagna, S. P. Ratti, V. Re, M. Ressegotti, C. Riccardi, P. Salvini, I. Vai, P. Vitulo, L. Alunni Solestizi, M. Biasini, G. M. Bilei, C. Cecchi, D. Ciangottini, L. Fanò, P. Lariccia, R. Leonardi, E. Manoni, G. Mantovani, V. Mariani, M. Menichelli, A. Rossi, A. Santocchia, D. Spiga, K. Androsov, P. Azzurri, G. Bagliesi, T. Boccali, L. Borrello, R. Castaldi, M. A. Ciocci, R. Dell’Orso, G. Fedi, L. Giannini, A. Giassi, M. T. Grippo, F. Ligabue, T. Lomtadze, E. Manca, G. Mandorli, L. Martini, A. Messineo, F. Palla, A. Rizzi, A. Savoy-Navarro, P. Spagnolo, R. Tenchini, G. Tonelli, A. Venturi, P. G. Verdini, L. Barone, F. Cavallari, M. Cipriani, D. Del Re, E. Di Marco, M. Diemoz, S. Gelli, E. Longo, F. Margaroli, B. Marzocchi, P. Meridiani, G. Organtini, R. Paramatti, F. Preiato, S. Rahatlou, C. Rovelli, F. Santanastasio, N. Amapane, R. Arcidiacono, S. Argiro, M. Arneodo, N. Bartosik, R. Bellan, C. Biino, N. Cartiglia, M. Costa, R. Covarelli, A. Degano, N. Demaria, B. Kiani, C. Mariotti, S. Maselli, G. Mazza, E. Migliore, V. Monaco, E. Monteil, M. Monteno, M. M. Obertino, L. Pacher, N. Pastrone, M. Pelliccioni, G. L. Pinna Angioni, F. Ravera, A. Romero, M. Ruspa, R. Sacchi, K. Shchelina, V. Sola, A. Solano, A. Staiano, P. Traczyk, S. Belforte, M. Casarsa, F. Cossutti, G. Della Ricca, A. Zanetti, D. H. Kim, G. N. Kim, M. S. Kim, J. Lee, S. Lee, S. W. Lee, C. S. Moon, Y. D. Oh, S. Sekmen, D. C. Son, Y. C. Yang, A. Lee, H. Kim, D. H. Moon, G. Oh, J. A. Brochero Cifuentes, J. Goh, T. J. Kim, S. Cho, S. Choi, Y. Go, D. Gyun, S. Ha, B. Hong, Y. Jo, Y. Kim, K. Lee, K. S. Lee, S. Lee, J. Lim, S. K. Park, Y. Roh, J. Almond, J. Kim, J. S. Kim, H. Lee, K. Lee, K. Nam, S. B. Oh, B. C. Radburn-Smith, S. h. Seo, U. K. Yang, H. D. Yoo, G. B. Yu, M. Choi, H. Kim, J. H. Kim, J. S. H. Lee, I. C. Park, Y. Choi, C. Hwang, J. Lee, I. Yu, V. Dudenas, A. Juodagalvis, J. Vaitkus, I. Ahmed, Z. A. Ibrahim, M. A. B. Md Ali, F. Mohamad Idris, W. A. T. Wan Abdullah, M. N. Yusli, Z. Zolkapli, H. Castilla-Valdez, E. De La Cruz-Burelo, M. C. Duran-Osuna, I. Heredia-De La Cruz, R. Lopez-Fernandez, J. Mejia Guisao, R. I. Rabadan-Trejo, G. Ramirez-Sanchez, R. Reyes-Almanza, A. Sanchez-Hernandez, S. Carrillo Moreno, C. Oropeza Barrera, F. Vazquez Valencia, I. Pedraza, H. A. Salazar Ibarguen, C. Uribe Estrada, A. Morelos Pineda, D. Krofcheck, P. H. Butler, A. Ahmad, M. Ahmad, Q. Hassan, H. R. Hoorani, A. Saddique, M. A. Shah, M. Shoaib, M. Waqas, H. Bialkowska, M. Bluj, B. Boimska, T. Frueboes, M. Górski, M. Kazana, K. Nawrocki, M. Szleper, P. Zalewski, K. Bunkowski, A. Byszuk, K. Doroba, A. Kalinowski, M. Konecki, J. Krolikowski, M. Misiura, M. Olszewski, A. Pyskir, M. Walczak, P. Bargassa, C. Beirão Da Cruz E Silva, A. Di Francesco, P. Faccioli, B. Galinhas, M. Gallinaro, J. Hollar, N. Leonardo, L. Lloret Iglesias, M. V. Nemallapudi, J. Seixas, G. Strong, O. Toldaiev, D. Vadruccio, J. Varela, S. Afanasiev, P. Bunin, M. Gavrilenko, I. Golutvin, I. Gorbunov, A. Kamenev, V. Karjavin, A. Lanev, A. Malakhov, V. Matveev, V. Palichik, V. Perelygin, S. Shmatov, S. Shulha, N. Skatchkov, V. Smirnov, N. Voytishin, A. Zarubin, Y. Ivanov, V. Kim, E. Kuznetsova, P. Levchenko, V. Murzin, V. Oreshkin, I. Smirnov, V. Sulimov, L. Uvarov, S. Vavilov, A. Vorobyev, Yu. Andreev, A. Dermenev, S. Gninenko, N. Golubev, A. Karneyeu, M. Kirsanov, N. Krasnikov, A. Pashenkov, D. Tlisov, A. Toropin, V. Epshteyn, V. Gavrilov, N. Lychkovskaya, V. Popov, I. Pozdnyakov, G. Safronov, A. Spiridonov, A. Stepennov, M. Toms, E. Vlasov, A. Zhokin, T. Aushev, A. Bylinkin, R. Chistov, M. Danilov, P. Parygin, D. Philippov, S. Polikarpov, E. Tarkovskii, V. Andreev, M. Azarkin, I. Dremin, M. Kirakosyan, A. Terkulov, A. Baskakov, A. Belyaev, E. Boos, M. Dubinin, L. Dudko, A. Ershov, A. Gribushin, V. Klyukhin, O. Kodolova, I. Lokhtin, I. Miagkov, S. Obraztsov, S. Petrushanko, V. Savrin, A. Snigirev, V. Blinov, Y. Skovpen, D. Shtol, I. Azhgirey, I. Bayshev, S. Bitioukov, D. Elumakhov, V. Kachanov, A. Kalinin, D. Konstantinov, P. Mandrik, V. Petrov, R. Ryutin, A. Sobol, S. Troshin, N. Tyurin, A. Uzunian, A. Volkov, P. Adzic, P. Cirkovic, D. Devetak, M. Dordevic, J. Milosevic, V. Rekovic, J. Alcaraz Maestre, A. Álvarez Fernández, M. Barrio Luna, M. Cerrada, N. Colino, B. De La Cruz, A. Delgado Peris, A. Escalante Del Valle, C. Fernandez Bedoya, J. P. Fernández Ramos, J. Flix, M. C. Fouz, P. Garcia-Abia, O. Gonzalez Lopez, S. Goy Lopez, J. M. Hernandez, M. I. Josa, D. Moran, A. Pérez-Calero Yzquierdo, J. Puerta Pelayo, A. Quintario Olmeda, I. Redondo, L. Romero, M. S. Soares, C. Albajar, J. F. de Trocóniz, M. Missiroli, J. Cuevas, C. Erice, J. Fernandez Menendez, I. Gonzalez Caballero, J. R. González Fernández, E. Palencia Cortezon, S. Sanchez Cruz, P. Vischia, J. M. Vizan Garcia, I. J. Cabrillo, A. Calderon, B. Chazin Quero, E. Curras, J. Duarte Campderros, M. Fernandez, J. Garcia-Ferrero, G. Gomez, A. Lopez Virto, J. Marco, C. Martinez Rivero, P. Martinez Ruiz del Arbol, F. Matorras, J. Piedra Gomez, T. Rodrigo, A. Ruiz-Jimeno, L. Scodellaro, N. Trevisani, I. Vila, R. Vilar Cortabitarte, D. Abbaneo, B. Akgun, E. Auffray, P. Baillon, A. H. Ball, D. Barney, M. Bianco, P. Bloch, A. Bocci, C. Botta, T. Camporesi, R. Castello, M. Cepeda, G. Cerminara, E. Chapon, Y. Chen, D. d’Enterria, A. Dabrowski, V. Daponte, A. David, M. De Gruttola, A. De Roeck, N. Deelen, M. Dobson, T. du Pree, M. Dünser, N. Dupont, A. Elliott-Peisert, P. Everaerts, F. Fallavollita, G. Franzoni, J. Fulcher, W. Funk, D. Gigi, A. Gilbert, K. Gill, F. Glege, D. Gulhan, P. Harris, J. Hegeman, V. Innocente, A. Jafari, P. Janot, O. Karacheban, J. Kieseler, V. Knünz, A. Kornmayer, M. J. Kortelainen, C. Lange, P. Lecoq, C. Lourenço, M. T. Lucchini, L. Malgeri, M. Mannelli, A. Martelli, F. Meijers, J. A. Merlin, S. Mersi, E. Meschi, P. Milenovic, F. Moortgat, M. Mulders, H. Neugebauer, J. Ngadiuba, S. Orfanelli, L. Orsini, L. Pape, E. Perez, M. Peruzzi, A. Petrilli, G. Petrucciani, A. Pfeiffer, M. Pierini, D. Rabady, A. Racz, T. Reis, G. Rolandi, M. Rovere, H. Sakulin, C. Schäfer, C. Schwick, M. Seidel, M. Selvaggi, A. Sharma, P. Silva, P. Sphicas, A. Stakia, J. Steggemann, M. Stoye, M. Tosi, D. Treille, A. Triossi, A. Tsirou, V. Veckalns, M. Verweij, W. D. Zeuner, W. Bertl, L. Caminada, K. Deiters, W. Erdmann, R. Horisberger, Q. Ingram, H. C. Kaestli, D. Kotlinski, U. Langenegger, T. Rohe, S. A. Wiederkehr, M. Backhaus, L. Bäni, P. Berger, L. Bianchini, B. Casal, G. Dissertori, M. Dittmar, M. Donegà, C. Dorfer, C. Grab, C. Heidegger, D. Hits, J. Hoss, G. Kasieczka, T. Klijnsma, W. Lustermann, B. Mangano, M. Marionneau, M. T. Meinhard, D. Meister, F. Micheli, P. Musella, F. Nessi-Tedaldi, F. Pandolfi, J. Pata, F. Pauss, G. Perrin, L. Perrozzi, M. Reichmann, D. A. Sanz Becerra, M. Schönenberger, L. Shchutska, V. R. Tavolaro, K. Theofilatos, M. L. Vesterbacka Olsson, R. Wallny, D. H. Zhu, T. K. Aarrestad, C. Amsler, M. F. Canelli, A. De Cosa, R. Del Burgo, S. Donato, C. Galloni, T. Hreus, B. Kilminster, D. Pinna, G. Rauco, P. Robmann, D. Salerno, K. Schweiger, C. Seitz, Y. Takahashi, A. Zucchetta, V. Candelise, T. H. Doan, Sh. Jain, R. Khurana, C. M. Kuo, W. Lin, A. Pozdnyakov, S. S. Yu, Arun Kumar, P. Chang, Y. Chao, K. F. Chen, P. H. Chen, F. Fiori, W.-S. Hou, Y. Hsiung, Y. F. Liu, R.-S. Lu, E. Paganis, A. Psallidas, A. Steen, J. F. Tsai, B. Asavapibhop, K. Kovitanggoon, G. Singh, N. Srimanobhas, F. Boran, S. Cerci, S. Damarseckin, Z. S. Demiroglu, C. Dozen, I. Dumanoglu, S. Girgis, G. Gokbulut, Y. Guler, I. Hos, E. E. Kangal, O. Kara, A. Kayis Topaksu, U. Kiminsu, M. Oglakci, G. Onengut, K. Ozdemir, D. Sunar Cerci, B. Tali, S. Turkcapar, I. S. Zorbakir, C. Zorbilmez, B. Bilin, G. Karapinar, K. Ocalan, M. Yalvac, M. Zeyrek, E. Gülmez, M. Kaya, O. Kaya, S. Tekten, E. A. Yetkin, M. N. Agaras, S. Atay, A. Cakir, K. Cankocak, B. Grynyov, L. Levchuk, F. Ball, L. Beck, J. J. Brooke, D. Burns, E. Clement, D. Cussans, O. Davignon, H. Flacher, J. Goldstein, G. P. Heath, H. F. Heath, J. Jacob, L. Kreczko, D. M. Newbold, S. Paramesvaran, T. Sakuma, S. Seif ElNasr-storey, D. Smith, V. J. Smith, K. W. Bell, A. Belyaev, C. Brew, R. M. Brown, L. Calligaris, D. Cieri, D. J. A. Cockerill, J. A. Coughlan, K. Harder, S. Harper, E. Olaiya, D. Petyt, C. H. Shepherd-Themistocleous, A. Thea, I. R. Tomalin, T. Williams, G. Auzinger, R. Bainbridge, J. Borg, S. Breeze, O. Buchmuller, A. Bundock, S. Casasso, M. Citron, D. Colling, L. Corpe, P. Dauncey, G. Davies, A. De Wit, M. Della Negra, R. Di Maria, A. Elwood, Y. Haddad, G. Hall, G. Iles, T. James, R. Lane, C. Laner, L. Lyons, A.-M. Magnan, S. Malik, L. Mastrolorenzo, T. Matsushita, J. Nash, A. Nikitenko, V. Palladino, M. Pesaresi, D. M. Raymond, A. Richards, A. Rose, E. Scott, C. Seez, A. Shtipliyski, S. Summers, A. Tapper, K. Uchida, M. Vazquez Acosta, T. Virdee, N. Wardle, D. Winterbottom, J. Wright, S. C. Zenz, J. E. Cole, P. R. Hobson, A. Khan, P. Kyberd, I. D. Reid, P. Symonds, L. Teodorescu, M. Turner, S. Zahid, A. Borzou, K. Call, J. Dittmann, K. Hatakeyama, H. Liu, N. Pastika, C. Smith, R. Bartek, A. Dominguez, A. Buccilli, S. I. Cooper, C. Henderson, P. Rumerio, C. West, D. Arcaro, A. Avetisyan, T. Bose, D. Gastler, D. Rankin, C. Richardson, J. Rohlf, L. Sulak, D. Zou, G. Benelli, D. Cutts, A. Garabedian, M. Hadley, J. Hakala, U. Heintz, J. M. Hogan, K. H. M. Kwok, E. Laird, G. Landsberg, J. Lee, Z. Mao, M. Narain, J. Pazzini, S. Piperov, S. Sagir, R. Syarif, D. Yu, R. Band, C. Brainerd, R. Breedon, D. Burns, M. Calderon De La Barca Sanchez, M. Chertok, J. Conway, R. Conway, P. T. Cox, R. Erbacher, C. Flores, G. Funk, M. Gardner, W. Ko, R. Lander, C. Mclean, M. Mulhearn, D. Pellett, J. Pilot, S. Shalhout, M. Shi, J. Smith, D. Stolp, K. Tos, M. Tripathi, Z. Wang, M. Bachtis, C. Bravo, R. Cousins, A. Dasgupta, A. Florent, J. Hauser, M. Ignatenko, N. Mccoll, S. Regnard, D. Saltzberg, C. Schnaible, V. Valuev, E. Bouvier, K. Burt, R. Clare, J. Ellison, J. W. Gary, S. M. A. Ghiasi Shirazi, G. Hanson, J. Heilman, E. Kennedy, F. Lacroix, O. R. Long, M. Olmedo Negrete, M. I. Paneva, W. Si, L. Wang, H. Wei, S. Wimpenny, B. R. Yates, J. G. Branson, S. Cittolin, M. Derdzinski, D. Gilbert, B. Hashemi, A. Holzner, D. Klein, G. Kole, V. Krutelyov, J. Letts, I. Macneill, M. Masciovecchio, D. Olivito, S. Padhi, M. Pieri, M. Sani, V. Sharma, S. Simon, M. Tadel, A. Vartak, S. Wasserbaech, J. Wood, F. Würthwein, A. Yagil, G. Zevi Della Porta, N. Amin, R. Bhandari, J. Bradmiller-Feld, C. Campagnari, A. Dishaw, V. Dutta, M. Franco Sevilla, C. George, F. Golf, L. Gouskos, J. Gran, R. Heller, J. Incandela, S. D. Mullin, A. Ovcharova, H. Qu, J. Richman, D. Stuart, I. Suarez, J. Yoo, D. Anderson, J. Bendavid, A. Bornheim, J. M. Lawhorn, H. B. Newman, T. Nguyen, C. Pena, M. Spiropulu, J. R. Vlimant, S. Xie, Z. Zhang, R. Y. Zhu, M. B. Andrews, T. Ferguson, T. Mudholkar, M. Paulini, J. Russ, M. Sun, H. Vogel, I. Vorobiev, M. Weinberg, J. P. Cumalat, W. T. Ford, F. Jensen, A. Johnson, M. Krohn, S. Leontsinis, T. Mulholland, K. Stenson, S. R. Wagner, J. Alexander, J. Chaves, J. Chu, S. Dittmer, K. Mcdermott, N. Mirman, J. R. Patterson, D. Quach, A. Rinkevicius, A. Ryd, L. Skinnari, L. Soffi, S. M. Tan, Z. Tao, J. Thom, J. Tucker, P. Wittich, M. Zientek, S. Abdullin, M. Albrow, M. Alyari, G. Apollinari, A. Apresyan, A. Apyan, S. Banerjee, L. A. T. Bauerdick, A. Beretvas, J. Berryhill, P. C. Bhat, G. Bolla, K. Burkett, J. N. Butler, A. Canepa, G. B. Cerati, H. W. K. Cheung, F. Chlebana, M. Cremonesi, J. Duarte, V. D. Elvira, J. Freeman, Z. Gecse, E. Gottschalk, L. Gray, D. Green, S. Grünendahl, O. Gutsche, R. M. Harris, S. Hasegawa, J. Hirschauer, Z. Hu, B. Jayatilaka, S. Jindariani, M. Johnson, U. Joshi, B. Klima, B. Kreis, S. Lammel, D. Lincoln, R. Lipton, M. Liu, T. Liu, R. Lopes De Sá, J. Lykken, K. Maeshima, N. Magini, J. M. Marraffino, D. Mason, P. McBride, P. Merkel, S. Mrenna, S. Nahn, V. O’Dell, K. Pedro, O. Prokofyev, G. Rakness, L. Ristori, B. Schneider, E. Sexton-Kennedy, A. Soha, W. J. Spalding, L. Spiegel, S. Stoynev, J. Strait, N. Strobbe, L. Taylor, S. Tkaczyk, N. V. Tran, L. Uplegger, E. W. Vaandering, C. Vernieri, M. Verzocchi, R. Vidal, M. Wang, H. A. Weber, A. Whitbeck, D. Acosta, P. Avery, P. Bortignon, D. Bourilkov, A. Brinkerhoff, A. Carnes, M. Carver, D. Curry, R. D. Field, I. K. Furic, J. Konigsberg, A. Korytov, K. Kotov, P. Ma, K. Matchev, H. Mei, G. Mitselmakher, D. Rank, D. Sperka, N. Terentyev, L. Thomas, J. Wang, S. Wang, J. Yelton, Y. R. Joshi, S. Linn, P. Markowitz, J. L. Rodriguez, A. Ackert, T. Adams, A. Askew, S. Hagopian, V. Hagopian, K. F. Johnson, T. Kolberg, G. Martinez, T. Perry, H. Prosper, A. Saha, A. Santra, V. Sharma, R. Yohay, M. M. Baarmand, V. Bhopatkar, S. Colafranceschi, M. Hohlmann, D. Noonan, T. Roy, F. Yumiceva, M. R. Adams, L. Apanasevich, D. Berry, R. R. Betts, R. Cavanaugh, X. Chen, O. Evdokimov, C. E. Gerber, D. A. Hangal, D. J. Hofman, K. Jung, J. Kamin, I. D. Sandoval Gonzalez, M. B. Tonjes, H. Trauger, N. Varelas, H. Wang, Z. Wu, J. Zhang, B. Bilki, W. Clarida, K. Dilsiz, S. Durgut, R. P. Gandrajula, M. Haytmyradov, V. Khristenko, J.-P. Merlo, H. Mermerkaya, A. Mestvirishvili, A. Moeller, J. Nachtman, H. Ogul, Y. Onel, F. Ozok, A. Penzo, C. Snyder, E. Tiras, J. Wetzel, K. Yi, B. Blumenfeld, A. Cocoros, N. Eminizer, D. Fehling, L. Feng, A. V. Gritsan, P. Maksimovic, J. Roskes, U. Sarica, M. Swartz, M. Xiao, C. You, A. Al-bataineh, P. Baringer, A. Bean, S. Boren, J. Bowen, J. Castle, S. Khalil, A. Kropivnitskaya, D. Majumder, W. Mcbrayer, M. Murray, C. Royon, S. Sanders, E. Schmitz, J. D. Tapia Takaki, Q. Wang, A. Ivanov, K. Kaadze, Y. Maravin, A. Mohammadi, L. K. Saini, N. Skhirtladze, S. Toda, F. Rebassoo, D. Wright, C. Anelli, A. Baden, O. Baron, A. Belloni, B. Calvert, S. C. Eno, Y. Feng, C. Ferraioli, N. J. Hadley, S. Jabeen, G. Y. Jeng, R. G. Kellogg, J. Kunkle, A. C. Mignerey, F. Ricci-Tam, Y. H. Shin, A. Skuja, S. C. Tonwar, D. Abercrombie, B. Allen, V. Azzolini, R. Barbieri, A. Baty, R. Bi, S. Brandt, W. Busza, I. A. Cali, M. D’Alfonso, Z. Demiragli, G. Gomez Ceballos, M. Goncharov, D. Hsu, M. Hu, Y. Iiyama, G. M. Innocenti, M. Klute, D. Kovalskyi, Y. S. Lai, Y.-J. Lee, A. Levin, P. D. Luckey, B. Maier, A. C. Marini, C. Mcginn, C. Mironov, S. Narayanan, X. Niu, C. Paus, C. Roland, G. Roland, J. Salfeld-Nebgen, G. S. F. Stephans, K. Tatar, D. Velicanu, J. Wang, T. W. Wang, B. Wyslouch, A. C. Benvenuti, R. M. Chatterjee, A. Evans, P. Hansen, J. Hiltbrand, S. Kalafut, Y. Kubota, Z. Lesko, J. Mans, S. Nourbakhsh, N. Ruckstuhl, R. Rusack, J. Turkewitz, M. A. Wadud, J. G. Acosta, S. Oliveros, E. Avdeeva, K. Bloom, D. R. Claes, C. Fangmeier, R. Gonzalez Suarez, R. Kamalieddin, I. Kravchenko, J. Monroy, J. E. Siado, G. R. Snow, B. Stieger, J. Dolen, A. Godshalk, C. Harrington, I. Iashvili, D. Nguyen, A. Parker, S. Rappoccio, B. Roozbahani, G. Alverson, E. Barberis, A. Hortiangtham, A. Massironi, D. M. Morse, T. Orimoto, R. Teixeira De Lima, D. Trocino, D. Wood, S. Bhattacharya, O. Charaf, K. A. Hahn, N. Mucia, N. Odell, B. Pollack, M. H. Schmitt, K. Sung, M. Trovato, M. Velasco, N. Dev, M. Hildreth, K. Hurtado Anampa, C. Jessop, D. J. Karmgard, N. Kellams, K. Lannon, N. Loukas, N. Marinelli, F. Meng, C. Mueller, Y. Musienko, M. Planer, A. Reinsvold, R. Ruchti, G. Smith, S. Taroni, M. Wayne, M. Wolf, A. Woodard, J. Alimena, L. Antonelli, B. Bylsma, L. S. Durkin, S. Flowers, B. Francis, A. Hart, C. Hill, W. Ji, B. Liu, W. Luo, D. Puigh, B. L. Winer, H. W. Wulsin, S. Cooperstein, O. Driga, P. Elmer, J. Hardenbrook, P. Hebda, S. Higginbotham, D. Lange, J. Luo, D. Marlow, K. Mei, I. Ojalvo, J. Olsen, C. Palmer, P. Piroué, D. Stickland, C. Tully, S. Malik, S. Norberg, A. Barker, V. E. Barnes, S. Das, S. Folgueras, L. Gutay, M. K. Jha, M. Jones, A. W. Jung, A. Khatiwada, D. H. Miller, N. Neumeister, C. C. Peng, H. Qiu, J. F. Schulte, J. Sun, F. Wang, W. Xie, T. Cheng, N. Parashar, J. Stupak, A. Adair, Z. Chen, K. M. Ecklund, S. Freed, F. J. M. Geurts, M. Guilbaud, M. Kilpatrick, W. Li, B. Michlin, M. Northup, B. P. Padley, J. Roberts, J. Rorie, W. Shi, Z. Tu, J. Zabel, A. Zhang, A. Bodek, P. de Barbaro, R. Demina, Y. T. Duh, T. Ferbel, M. Galanti, A. Garcia-Bellido, J. Han, O. Hindrichs, A. Khukhunaishvili, K. H. Lo, P. Tan, M. Verzetti, R. Ciesielski, K. Goulianos, C. Mesropian, A. Agapitos, J. P. Chou, Y. Gershtein, T. A. Gómez Espinosa, E. Halkiadakis, M. Heindl, E. Hughes, S. Kaplan, R. Kunnawalkam Elayavalli, S. Kyriacou, A. Lath, R. Montalvo, K. Nash, M. Osherson, H. Saka, S. Salur, S. Schnetzer, D. Sheffield, S. Somalwar, R. Stone, S. Thomas, P. Thomassen, M. Walker, A. G. Delannoy, M. Foerster, J. Heideman, G. Riley, K. Rose, S. Spanier, K. Thapa, O. Bouhali, A. Castaneda Hernandez, A. Celik, M. Dalchenko, M. De Mattia, A. Delgado, S. Dildick, R. Eusebi, J. Gilmore, T. Huang, T. Kamon, R. Mueller, Y. Pakhotin, R. Patel, A. Perloff, L. Perniè, D. Rathjens, A. Safonov, A. Tatarinov, K. A. Ulmer, N. Akchurin, J. Damgov, F. De Guio, P. R. Dudero, J. Faulkner, E. Gurpinar, S. Kunori, K. Lamichhane, S. W. Lee, T. Libeiro, T. Peltola, S. Undleeb, I. Volobouev, Z. Wang, S. Greene, A. Gurrola, R. Janjam, W. Johns, C. Maguire, A. Melo, H. Ni, K. Padeken, P. Sheldon, S. Tuo, J. Velkovska, Q. Xu, M. W. Arenton, P. Barria, B. Cox, R. Hirosky, M. Joyce, A. Ledovskoy, H. Li, C. Neu, T. Sinthuprasith, Y. Wang, E. Wolfe, F. Xia, R. Harr, P. E. Karchin, N. Poudyal, J. Sturdy, P. Thapa, S. Zaleski, M. Brodski, J. Buchanan, C. Caillol, S. Dasu, L. Dodd, S. Duric, B. Gomber, M. Grothe, M. Herndon, A. Hervé, U. Hussain, P. Klabbers, A. Lanaro, A. Levine, K. Long, R. Loveless, G. Polese, T. Ruggles, A. Savin, N. Smith, W. H. Smith, D. Taylor, N. Woods, CMS Collaboration

**Affiliations:** 1 Yerevan Physics Institute, Yerevan, Armenia; 2 Institut für Hochenergiephysik, Wien, Austria; 3 Institute for Nuclear Problems, Minsk, Belarus; 4 Universiteit Antwerpen, Antwerpen, Belgium; 5 Vrije Universiteit Brussel, Brussels, Belgium; 6 Université Libre de Bruxelles, Brussels, Belgium; 7 Ghent University, Ghent, Belgium; 8 Université Catholique de Louvain, Louvain-la-Neuve, Belgium; 9 Université de Mons, Mons, Belgium; 10 Centro Brasileiro de Pesquisas Fisicas, Rio de Janeiro, Brazil; 11 Universidade do Estado do Rio de Janeiro, Rio de Janeiro, Brazil; 12 Universidade Estadual Paulista, Universidade Federal do ABC, São Paulo, Brazil; 13 Institute for Nuclear Research and Nuclear Energy, Bulgarian Academy of Sciences, Sofia, Bulgaria; 14 University of Sofia, Sofia, Bulgaria; 15 Beihang University, Beijing, China; 16 Institute of High Energy Physics, Beijing, China; 17 State Key Laboratory of Nuclear Physics and Technology, Peking University, Beijing, China; 18 Universidad de Los Andes, Bogotá, Colombia; 19 University of Split, Faculty of Electrical Engineering, Mechanical Engineering and Naval Architecture, Split, Croatia; 20 University of Split, Faculty of Science, Split, Croatia; 21 Institute Rudjer Boskovic, Zagreb, Croatia; 22 University of Cyprus, Nicosia, Cyprus; 23 Charles University, Prague, Czech Republic; 24 Universidad San Francisco de Quito, Quito, Ecuador; 25 Academy of Scientific Research and Technology of the Arab Republic of Egypt, Egyptian Network of High Energy Physics, Cairo, Egypt; 26 National Institute of Chemical Physics and Biophysics, Tallinn, Estonia; 27 Department of Physics, University of Helsinki, Helsinki, Finland; 28 Helsinki Institute of Physics, Helsinki, Finland; 29 Lappeenranta University of Technology, Lappeenranta, Finland; 30 IRFU, CEA, Université Paris-Saclay, Gif-sur-Yvette, France; 31 Laboratoire Leprince-Ringuet, Ecole polytechnique, CNRS/IN2P3, Université Paris-Saclay, Palaiseau, France; 32 Université de Strasbourg, CNRS, IPHC UMR 7178, 67000 Strasbourg, France; 33 Centre de Calcul de l’Institut National de Physique Nucleaire et de Physique des Particules, CNRS/IN2P3, Villeurbanne, France; 34 Université de Lyon, Université Claude Bernard Lyon 1, CNRS-IN2P3, Institut de Physique Nucléaire de Lyon, Villeurbanne, France; 35 Georgian Technical University, Tbilisi, Georgia; 36 Tbilisi State University, Tbilisi, Georgia; 37 RWTH Aachen University, I. Physikalisches Institut, Aachen, Germany; 38 RWTH Aachen University, III. Physikalisches Institut A, Aachen, Germany; 39 RWTH Aachen University, III. Physikalisches Institut B, Aachen, Germany; 40 Deutsches Elektronen-Synchrotron, Hamburg, Germany; 41 University of Hamburg, Hamburg, Germany; 42 Institut für Experimentelle Kernphysik, Karlsruhe, Germany; 43 Institute of Nuclear and Particle Physics (INPP), NCSR Demokritos, Aghia Paraskevi, Greece; 44 National and Kapodistrian University of Athens, Athens, Greece; 45 National Technical University of Athens, Athens, Greece; 46 University of Ioánnina, Ioannina, Greece; 47 MTA-ELTE Lendület CMS Particle and Nuclear Physics Group, Eötvös Loránd University, Budapest, Hungary; 48 Wigner Research Centre for Physics, Budapest, Hungary; 49 Institute of Nuclear Research ATOMKI, Debrecen, Hungary; 50 Institute of Physics, University of Debrecen, Debrecen, Hungary; 51 Indian Institute of Science (IISc), Bangalore, India; 52 National Institute of Science Education and Research, Bhubaneswar, India; 53 Panjab University, Chandigarh, India; 54 University of Delhi, Delhi, India; 55 Saha Institute of Nuclear Physics, HBNI, Kolkata, India; 56 Indian Institute of Technology Madras, Madras, India; 57 Bhabha Atomic Research Centre, Mumbai, India; 58 Tata Institute of Fundamental Research-A, Mumbai, India; 59 Tata Institute of Fundamental Research-B, Mumbai, India; 60 Indian Institute of Science Education and Research (IISER), Pune, India; 61 Institute for Research in Fundamental Sciences (IPM), Tehran, Iran; 62 University College Dublin, Dublin, Ireland; 63 INFN Sezione di Bari, Università di Bari, Politecnico di Bari, Bari, Italy; 64 INFN Sezione di Bologna, Università di Bologna, Bologna, Italy; 65 INFN Sezione di Catania, Università di Catania, Catania, Italy; 66 INFN Sezione di Firenze, Università di Firenze, Florence, Italy; 67 INFN Laboratori Nazionali di Frascati, Frascati, Italy; 68 INFN Sezione di Genova, Università di Genova, Genoa, Italy; 69 INFN Sezione di Milano-Bicocca, Università di Milano-Bicocca, Milan, Italy; 70 INFN Sezione di Napoli, Università di Napoli ’Federico II’ , Naples , Italy, Università della Basilicata, Potenza, Italy, Università G. Marconi, Rome, Italy; 71 INFN Sezione di Padova, Università di Padova, Padova, Italy, Università di Trento, Trento, Italy; 72 INFN Sezione di Pavia, Università di Pavia, Pavia, Italy; 73 INFN Sezione di Perugia, Università di Perugia, Perugia, Italy; 74 INFN Sezione di Pisa, Università di Pisa, Scuola Normale Superiore di Pisa, Pisa, Italy; 75 INFN Sezione di Roma, Sapienza Università di Roma, Rome, Italy; 76 INFN Sezione di Torino, Università di Torino, Turin, Italy, Università del Piemonte Orientale, Novara, Italy; 77 INFN Sezione di Trieste, Università di Trieste, Trieste, Italy; 78 Kyungpook National University, Daegu, Korea; 79 Chonbuk National University, Jeonju, Korea; 80 Chonnam National University, Institute for Universe and Elementary Particles, Kwangju, Korea; 81 Hanyang University, Seoul, Korea; 82 Korea University, Seoul, Korea; 83 Seoul National University, Seoul, Korea; 84 University of Seoul, Seoul, Korea; 85 Sungkyunkwan University, Suwon, Korea; 86 Vilnius University, Vilnius, Lithuania; 87 National Centre for Particle Physics, Universiti Malaya, Kuala Lumpur, Malaysia; 88 Centro de Investigacion y de Estudios Avanzados del IPN, Mexico City, Mexico; 89 Universidad Iberoamericana, Mexico City, Mexico; 90 Benemerita Universidad Autonoma de Puebla, Puebla, Mexico; 91 Universidad Autónoma de San Luis Potosí, San Luis Potosí, Mexico; 92 University of Auckland, Auckland, New Zealand; 93 University of Canterbury, Christchurch, New Zealand; 94 National Centre for Physics, Quaid-I-Azam University, Islamabad, Pakistan; 95 National Centre for Nuclear Research, Swierk, Poland; 96 Institute of Experimental Physics, Faculty of Physics, University of Warsaw, Warsaw, Poland; 97 Laboratório de Instrumentação e Física Experimental de Partículas, Lisbon, Portugal; 98 Joint Institute for Nuclear Research, Dubna, Russia; 99 Petersburg Nuclear Physics Institute, Gatchina, (St. Petersburg), Russia; 100 Institute for Nuclear Research, Moscow, Russia; 101 Institute for Theoretical and Experimental Physics, Moscow, Russia; 102 Moscow Institute of Physics and Technology, Moscow, Russia; 103 National Research Nuclear University ‘Moscow Engineering Physics Institute’ (MEPhI), Moscow, Russia; 104 P.N. Lebedev Physical Institute, Moscow, Russia; 105 Skobeltsyn Institute of Nuclear Physics, Lomonosov Moscow State University, Moscow, Russia; 106 Novosibirsk State University (NSU), Novosibirsk, Russia; 107 State Research Center of Russian Federation, Institute for High Energy Physics, Protvino, Russia; 108 University of Belgrade, Faculty of Physics and Vinca Institute of Nuclear Sciences, Belgrade, Serbia; 109 Centro de Investigaciones Energéticas Medioambientales y Tecnológicas (CIEMAT), Madrid, Spain; 110 Universidad Autónoma de Madrid, Madrid, Spain; 111 Universidad de Oviedo, Oviedo, Spain; 112 Instituto de Física de Cantabria (IFCA), CSIC-Universidad de Cantabria, Santander, Spain; 113 CERN, European Organization for Nuclear Research, Geneva, Switzerland; 114 Paul Scherrer Institut, Villigen, Switzerland; 115 ETH Zurich-Institute for Particle Physics and Astrophysics (IPA), Zurich, Switzerland; 116 Universität Zürich, Zurich, Switzerland; 117 National Central University, Chung-Li, Taiwan; 118 National Taiwan University (NTU), Taipei, Taiwan; 119 Chulalongkorn University, Faculty of Science, Department of Physics, Bangkok, Thailand; 120 Çukurova University, Physics Department, Science and Art Faculty, Adana, Turkey; 121 Middle East Technical University, Physics Department, Ankara, Turkey; 122 Bogazici University, Istanbul, Turkey; 123 Istanbul Technical University, Istanbul, Turkey; 124 Institute for Scintillation Materials of National Academy of Science of Ukraine, Kharkov, Ukraine; 125 National Scientific Center, Kharkov Institute of Physics and Technology, Kharkov, Ukraine; 126 University of Bristol, Bristol, UK; 127 Rutherford Appleton Laboratory, Didcot, UK; 128 Imperial College, London, UK; 129 Brunel University, Uxbridge, UK; 130 Baylor University, Waco, USA; 131 Catholic University of America, Washington, DC, USA; 132 The University of Alabama, Tuscaloosa, USA; 133 Boston University, Boston, USA; 134 Brown University, Providence, USA; 135 University of California, Davis, Davis, USA; 136 University of California, Los Angeles, USA; 137 University of California, Riverside, Riverside, USA; 138 University of California, San Diego, La Jolla, USA; 139 University of California, Santa Barbara-Department of Physics, Santa Barbara, USA; 140 California Institute of Technology, Pasadena, USA; 141 Carnegie Mellon University, Pittsburgh, USA; 142 University of Colorado Boulder, Boulder, USA; 143 Cornell University, Ithaca, USA; 144 Fermi National Accelerator Laboratory, Batavia, USA; 145 University of Florida, Gainesville, USA; 146 Florida International University, Miami, USA; 147 Florida State University, Tallahassee, USA; 148 Florida Institute of Technology, Melbourne, USA; 149 University of Illinois at Chicago (UIC), Chicago, USA; 150 The University of Iowa, Iowa City, USA; 151 Johns Hopkins University, Baltimore, USA; 152 The University of Kansas, Lawrence, USA; 153 Kansas State University, Manhattan, USA; 154 Lawrence Livermore National Laboratory, Livermore, USA; 155 University of Maryland, College Park, USA; 156 Massachusetts Institute of Technology, Cambridge, USA; 157 University of Minnesota, Minneapolis, USA; 158 University of Mississippi, Oxford, USA; 159 University of Nebraska-Lincoln, Lincoln, USA; 160 State University of New York at Buffalo, Buffalo, USA; 161 Northeastern University, Boston, USA; 162 Northwestern University, Evanston, USA; 163 University of Notre Dame, Notre Dame, USA; 164 The Ohio State University, Columbus, USA; 165 Princeton University, Princeton, USA; 166 University of Puerto Rico, Mayaguez, USA; 167 Purdue University, West Lafayette, USA; 168 Purdue University Northwest, Hammond, USA; 169 Rice University, Houston, USA; 170 University of Rochester, Rochester, USA; 171 The Rockefeller University, New York, USA; 172 Rutgers, The State University of New Jersey, Piscataway, USA; 173 University of Tennessee, Knoxville, USA; 174 Texas A & M University, College Station, USA; 175 Texas Tech University, Lubbock, USA; 176 Vanderbilt University, Nashville, USA; 177 University of Virginia, Charlottesville, USA; 178 Wayne State University, Detroit, USA; 179 University of Wisconsin-Madison, Madison, WI USA; 180 CERN, 1211 Geneva 23, Switzerland

## Abstract

Measurements are presented of the lifetimes of the ${\mathrm {B}^0}$, $\mathrm {B}^0_\mathrm {s}$, $\mathrm {\Lambda }_\mathrm {b} ^0$, and $\mathrm {B}_\mathrm {c}^+$ hadrons using the decay channels ${\mathrm {B}^0}\!\rightarrow \! \mathrm {J}/\psi \mathrm {K^{*}(892)}{}^{0}$, ${\mathrm {B}^0}\!\rightarrow \! \mathrm {J}/\psi \mathrm {K^0_S}$, $\mathrm {B}^0_\mathrm {s} \!\rightarrow \! \mathrm {J}/\psi \mathrm {\pi ^+}\mathrm {\pi ^-}$, $\mathrm {B}^0_\mathrm {s} \!\rightarrow \! \mathrm {J}/\psi \mathrm {\phi (1020)}$, $\varLambda _\mathrm {b}^0\!\rightarrow \!\mathrm {J}/\psi \mathrm {\Lambda }^{0}$, and $\mathrm {B}_\mathrm {c}^+ \!\rightarrow \!\mathrm {J}/\psi \mathrm {\pi ^+}$. The data sample, corresponding to an integrated luminosity of 19.7$\,\text {fb}^\text {-1}$, was collected by the CMS detector at the LHC in proton–proton collisions at $\sqrt{s}=8\,\text {Te}\text {V}$. The ${\mathrm {B}^0}$ lifetime is measured to be $453.0 \pm 1.6\,\text {(stat)} \pm 1.8\,\text {(syst)} \,\upmu \text {m}$ in $\mathrm {J}/\psi \mathrm {K^{*}(892)}{}^{0}$and $457.8 \pm 2.7\,\text {(stat)} \pm 2.8\,\text {(syst)} \,\upmu \text {m}$ in $\mathrm {J}/\psi \mathrm {K^0_S}$, which results in a combined measurement of $c\tau _{{\mathrm {B}^0}} = 454.1 \pm 1.4\,\text {(stat)} \pm 1.7\,\text {(syst)} \,\upmu \text {m}$. The effective lifetime of the $\mathrm {B}^0_\mathrm {s}$ meson is measured in two decay modes, with contributions from different amounts of the heavy and light eigenstates. This results in two different measured lifetimes: $c\tau _{\mathrm {B}^0_\mathrm {s} \rightarrow \mathrm {J}/\psi \mathrm {\pi ^+}\mathrm {\pi ^-}} = 502.7 \pm 10.2\,\text {(stat)} \pm 3.4\,\text {(syst)} \,\upmu \text {m}$ and $c\tau _{\mathrm {B}^0_\mathrm {s} \rightarrow \mathrm {J}/\psi \mathrm {\phi (1020)}} = 443.9 \pm 2.0\,\text {(stat)} \pm 1.5\,\text {(syst)} \,\upmu \text {m}$. The $\mathrm {\Lambda }_\mathrm {b} ^0$ lifetime is found to be $442.9 \pm 8.2\,\text {(stat)} \pm 2.8\,\text {(syst)} \,\upmu \text {m}$. The precision from each of these channels is as good as or better than previous measurements. The $\mathrm {B}_\mathrm {c}^+$ lifetime, measured with respect to the ${\mathrm {B}^{+}}$ to reduce the systematic uncertainty, is $162.3 \pm 7.8\,\text {(stat)} \pm 4.2\,\text {(syst)} \pm 0.1\,(\tau _{{\mathrm {B}^{+}}})\,\upmu \text {m}$. All results are in agreement with current world-average values.

## Introduction

Precise lifetime measurements involving the weak interaction play an important role in the study of nonperturbative aspects of quantum chromodynamics (QCD). The phenomenology is commonly described by the QCD-inspired heavy-quark expansion model, which provides estimates of the ratio of lifetimes for hadrons containing a common heavy quark [1]. In this paper, we report measurements of the lifetimes of the ${\mathrm {B}^0}$, $\mathrm {B}^0_\mathrm {s}$, $\varLambda _\mathrm {b}^0$, and $\mathrm {B}_\mathrm {c}^+$ hadrons.

The measurements are based on the reconstruction of the transverse decay length $L_{xy}$, where $\vec {L}_{xy}$ is defined as the flight distance vector from the primary vertex to the decay vertex of the $\mathrm {b}$ hadron, projected onto the transverse component $\vec {p}_\text {T}$ (perpendicular to the beam axis) of the $\mathrm {b}$ hadron momentum. The proper decay time of the $\mathrm {b}$ hadron times the speed of light is measured using

1 $$\begin{aligned} ct =c L_{xy}\frac{M}{p_{\mathrm {T}}}, \end{aligned}$$

where *M* is the world-average value of the mass of the $\mathrm {b}$ hadron [2].

In this analysis, the $\mathrm {b}$ hadrons are reconstructed from decays containing a $\mathrm {J}/\psi$ meson. The data were recorded by the CMS detector [3] at the CERN LHC using dedicated triggers that require two oppositely charged muons consistent with originating from a common vertex and with an invariant mass compatible with that of the $\mathrm {J}/\psi$ meson. Specifically, we reconstruct the decay modes ${\mathrm {B}^0}\!\rightarrow \! \mathrm {J}/\psi \mathrm {K^{*}(892)}{}^{0}$, ${\mathrm {B}^0}\!\rightarrow \! \mathrm {J}/\psi \mathrm {K^0_S}$, $\mathrm {B}^0_\mathrm {s} \!\rightarrow \! \mathrm {J}/\psi \mathrm {\pi ^+}\mathrm {\pi ^-}$, $\mathrm {B}^0_\mathrm {s} \!\rightarrow \! \mathrm {J}/\psi \mathrm {\phi (1020)}$, $\varLambda _\mathrm {b}^0\!\rightarrow \!\mathrm {J}/\psi \mathrm {\Lambda }^{0}$, and $\mathrm {B}_\mathrm {c}^+ \!\rightarrow \!\mathrm {J}/\psi \mathrm {\pi ^+}$, where $\mathrm {J}/\psi \!\rightarrow \! \upmu ^+ \upmu ^-$, $\mathrm {K^{*}(892)}{}^{0} \!\rightarrow \! \mathrm {K^+}\mathrm {\pi ^-}$, $\mathrm {K^0_S}\!\rightarrow \! \mathrm {\pi ^+}\mathrm {\pi ^-}$, $\mathrm {\phi (1020)}\!\rightarrow \! \mathrm {K^+}\mathrm {K^-}$, and $\mathrm {\Lambda }^{0}\!\rightarrow \! \mathrm {p}\mathrm {\pi ^-}$. The ${\mathrm {B}^{+}}\!\rightarrow \!\mathrm {J}/\psi \mathrm {K^+}$ decay is used as a reference mode and in evaluating some of the systematic uncertainties. Charge conjugation is implied throughout, unless otherwise indicated.

The decay rate of neutral $\mathrm {B}_{\mathrm {q}}$ ($\hbox {q} = \hbox {s or d}$) mesons is characterized by two parameters: the average decay width $\varGamma _{\mathrm {q}} = (\varGamma _{\mathrm {L}}^{\mathrm {q}} + \varGamma _{\mathrm {H}}^{\mathrm {q}})/2$ and the decay width difference $\varDelta \varGamma _{\mathrm {q}} = \varGamma _{\mathrm {L}}^{\mathrm {q}} - \varGamma _{\mathrm {H}}^{\mathrm {q}}$, where $\varGamma _{\mathrm {L,H}}^{\mathrm {q}}$ are the decay widths of the light (L) and heavy (H) mass eigenstates. Assuming equal amounts of $\mathrm {B}_{\mathrm {q}}$ and its antiparticle are produced in the proton–proton collisions, the time-dependent decay rate into a final state *f* that is accessible by both particle and antiparticle can be written as [4]:

2 $$\begin{aligned} R_{\mathrm {L}}^{f} \mathrm {e}^{- \varGamma _{\mathrm {L}}^{\mathrm {q}} t } + R_{\mathrm {H}}^{f} \mathrm {e}^{- \varGamma _{\mathrm {H}}^{\mathrm {q}} t}, \end{aligned}$$

where $R_{\mathrm {L}}^{f}$ and $R_{\mathrm {H}}^{f}$ are the amplitudes of the light and heavy mass states, respectively. Since the neutral B mesons have two eigenstates with different lifetimes, the $ct$ distribution consists of the sum of two exponential contributions. The effective lifetime of the neutral $\mathrm {B}_{\mathrm {q}}$ meson, produced as an equal admixture of particle and antiparticle flavour eigenstates and decaying into a final state *f*, can be written as [4]:

3 $$\begin{aligned} \tau _\text {eff} = \frac{ \frac{ R_\mathrm {L}^f}{ \left( \varGamma _{\mathrm {L}}^{\mathrm {q}} \right) ^2 } + \frac{R_\mathrm {H}^f}{ \left( \varGamma _\mathrm {H}^{\mathrm {q}} \right) ^2 }}{ \frac{R_\mathrm {L}^f}{\varGamma _{\mathrm {L}}^{\mathrm {q}} } + \frac{R_\mathrm {H}^f}{\varGamma _{\mathrm {H}}^{\mathrm {q}} } }. \end{aligned}$$

Since the amplitudes $R_{\mathrm {H}}^f$ and $R_{\mathrm {L}}^f$ are specific to the decay channel, the effective lifetime depends on the final state *f* and is measured by fitting an exponential function to a distribution consisting of the sum of two exponential contributions. Because the ${\mathrm {B}^0}$ system has a small lifetime difference with respect to the average lifetime, $\varDelta \varGamma _\mathrm {d}/\varGamma _\mathrm {d} = (-0.2 \pm 1.0)\%$ [5], the $ct$ distribution is close to an exponential, and it is treated as such for the lifetime measurement. Following Ref. [6], the ${\mathrm {B}^0}$ lifetimes measured in the flavour-specific channel ${\mathrm {B}^0}\!\rightarrow \! \mathrm {J}/\psi \mathrm {K^{*}(892)}{}^{0}$ and the *CP* eigenstate channel ${\mathrm {B}^0}\!\rightarrow \! \mathrm {J}/\psi \mathrm {K^0_S}$ are used to determine values for $\varDelta \varGamma _\mathrm {d}$, $\varGamma _\mathrm {d}$, and $\varDelta \varGamma _\mathrm {d}/\varGamma _\mathrm {d}$.

In the $\mathrm {B}^0_\mathrm {s}$ system, $\varDelta \varGamma _\mathrm {s}/\varGamma _{\mathrm {s}} = (13.0 \pm 0.9)\%$ [5] and the deviation from an exponential $ct$ distribution is sizeable. In this analysis, the two lifetimes associated with the $\mathrm {B}^0_\mathrm {s}$ meson are measured in the $\mathrm {J}/\psi \mathrm {\pi ^+}\mathrm {\pi ^-}$ and $\mathrm {J}/\psi \mathrm {\phi (1020)}$ decay channels. The $\mathrm {B}^0_\mathrm {s} \!\rightarrow \! \mathrm {J}/\psi \mathrm {\pi ^+}\mathrm {\pi ^-}$ decays are reconstructed in the invariant mass range $0.9240< M( \pi ^{+}\pi ^{-} ) < 1.0204$ $\,\text {GeV}$, which is dominated by the $f_{0}(980)$ resonance [7, 8], making it a CP-odd final state. Therefore, the lifetime measured in this channel is related to the inverse of the decay width of the heavy $\mathrm {B}^0_\mathrm {s}$ mass eigenstate, $\tau _{\mathrm {B}^0_\mathrm {s}}^{\text {CP-odd}} \approx 1/\varGamma _\mathrm {H}$, as CP violation in mixing is measured to be negligible [2]. The $\mathrm {J}/\psi \mathrm {\phi (1020)}$ decay channel is an admixture of CP-even and CP-odd states, corresponding to the light and heavy mass eigenstates, respectively, neglecting CP violation in mixing. Rewriting Eq. (3), the effective lifetime of the $\mathrm {B}^0_\mathrm {s}$ meson decaying to $\mathrm {J}/\psi \mathrm {\phi (1020)}$ can be expressed as

4 $$\begin{aligned} \tau _{\text {eff}} = f_\mathrm {H} \tau _\mathrm {H} + (1-f_\mathrm {H}) \tau _\mathrm {L}, \end{aligned}$$

where $\tau _\mathrm {L}$ and $\tau _\mathrm {H}$ are the lifetimes of the light and heavy mass states, respectively, and $f_\mathrm {H}$ is the heavy-component fraction, defined as:

5 $$\begin{aligned} f_\mathrm {H} = \frac{| A_{\perp } |^2 \tau _\mathrm {H}}{ |A|^2 \tau _\mathrm {L} + |A_\perp |^2 \tau _\mathrm {H}}. \end{aligned}$$

Here, $|A|^2 = | A_0(0) |^2 + | A_{\parallel }(0) |^2$ is the sum of the squares of the amplitudes of the two CP-even states, and $| A_{\perp } |^2 = | A_{\perp }(0) |^2$ is the square of the amplitude of the CP-odd state. The amplitudes are determined at the production time $t=0$. Normalization constraints require $|A|^2 = 1 - |A_\perp |^2$ and therefore

6 $$\begin{aligned} f_\mathrm {H} = \frac{| A_{\perp } |^2 \tau _\mathrm {H}}{ (1 - |A_\perp |^2) \tau _\mathrm {L} + |A_\perp |^2 \tau _\mathrm {H}}. \end{aligned}$$

By combining the $\mathrm {B}^0_\mathrm {s}$ lifetimes obtained from the final states $\mathrm {J}/\psi \mathrm {\phi (1020)}$ and $\mathrm {J}/\psi \mathrm {\pi ^+}\mathrm {\pi ^-}$, it is possible to determine the lifetime of the light $\mathrm {B}^0_\mathrm {s}$ mass eigenstate. The results in this paper are complementary to the CMS weak mixing phase analysis in the $\mathrm {B}^0_\mathrm {s} \!\rightarrow \! \mathrm {J}/\psi \mathrm {\phi (1020)}$ channel [9], which provided measurements of the average decay width $\varGamma _\mathrm {s}$ and the decay width difference $\varDelta \varGamma _\mathrm {s}$.

The weak decay of the $\mathrm {B}_\mathrm {c}^+$ meson can occur through either the b or c quark decaying, with the other quark as a spectator, or through an annihilation process. The latter is predicted to contribute 10% of the decay width [10], and lifetime measurements can be used to test the $\mathrm {B}_\mathrm {c}^+$ decay model. As fewer and less precise measurements of the $\mathrm {B}_\mathrm {c}^+$ lifetime exist [11–16] compared to other b hadrons, the $\mathrm {B}_\mathrm {c}^+$ lifetime measurement presented in this paper is particularly valuable.

## The CMS detector

The central feature of the CMS apparatus is a superconducting solenoid of 6 $\text {m}$ internal diameter, providing a magnetic field of 3.8 $\text {T}$. Within the solenoid volume are a silicon pixel and strip tracker, a lead tungstate crystal electromagnetic calorimeter, and a brass and scintillator hadron calorimeter, each composed of a barrel and two endcap sections. Forward calorimeters extend the pseudorapidity coverage provided by the barrel and endcap detectors. Muons are detected in gas-ionization chambers embedded in the steel flux-return yoke outside the solenoid.

The main subdetectors used for this analysis are the silicon tracker and the muon detection system. The silicon tracker measures charged particles in the pseudorapidity range $|\eta |<2.5$. It consists of 1440 silicon pixel and 15 148 silicon strip detector modules. For charged particles of $1< p_{\mathrm {T}} < 10\,\text {GeV}$ and $|\eta | < 1.4$, the track resolutions are typically 1.5% in $p_{\mathrm {T}}$ and 25–90 (45–150)$\,\upmu \text {m}$ in the transverse (longitudinal) impact parameter [17]. Muons are measured in the pseudorapidity range $|\eta | < 2.4$, with detection planes made using three technologies: drift tubes, cathode strip chambers, and resistive-plate chambers.

Events of interest are selected using a two-tiered trigger system [18]. The first level, composed of custom hardware processors, uses information from the calorimeters and muon detectors to select events at a rate of around 100 $\text {kHz}$ within a time interval of less than 4$\,\upmu \text {s}$. The second level, known as the high-level trigger (HLT), consists of a farm of processors running a version of the full event reconstruction software optimized for fast processing, and reduces the event rate to around 1 $\text {kHz}$ before data storage. At the HLT stage, there is full access to the event information, and therefore selection criteria similar to those applied offline can be used.

A more detailed description of the CMS detector, together with a definition of the coordinate system used and the relevant kinematic variables, can be found in Ref. [3].

## Data and Monte Carlo simulated samples

The data used in this analysis were collected in 2012 from proton–proton collisions at a centre-of-mass energy of 8$\,\text {Te}\text {V}$, and correspond to an integrated luminosity of 19.7$\,\text {fb}^\text {-1}$.

Fully simulated Monte Carlo (MC) samples of ${\mathrm {B}^{+}}\!\rightarrow \! \mathrm {J}/\psi \mathrm {K^+}$, ${\mathrm {B}^0}\!\rightarrow \! \mathrm {J}/\psi \mathrm {K^{*}(892)}{}^{0}$, ${\mathrm {B}^0}\!\rightarrow \! \mathrm {J}/\psi \mathrm {K^0_S}$, $\mathrm {B}^0_\mathrm {s} \!\rightarrow \! \mathrm {J}/\psi \mathrm {\pi ^+}\mathrm {\pi ^-}$, $\mathrm {B}^0_\mathrm {s} \!\rightarrow \! \mathrm {J}/\psi \mathrm {\phi (1020)}$, and $\varLambda _\mathrm {b}^0\!\rightarrow \!\mathrm {J}/\psi \mathrm {\Lambda }^{0}$ were produced with pythia 6.424 [19] to simulate the proton–proton collisions, and subsequent parton shower and hadronization processes. The $\mathrm {B}_\mathrm {c}^+$ MC sample was produced with the dedicated generator bcvegpy 2.0 [20, 21] interfaced to pythia . Decays of particles containing b or c quarks are simulated with the evtgen package [22], and final-state radiation is included via photos  [23]. Events are passed through the CMS detector simulation based on Geant4 [24], including additional proton–proton collisions in the same or nearby beam crossings (pileup) to match the number of multiple vertices per event in the data. Simulated events are processed with the same reconstruction and trigger algorithms as the data.

## Reconstruction of $\mathrm {b}$ hadrons

The data are collected with a trigger that is designed to identify events in which a $\mathrm {J}/\psi$ meson decays to two oppositely charged muons. The transverse momentum of the $\mathrm {J}/\psi$ candidate is required to be greater than 7.9$\,\text {GeV}$ and both muons must be in the pseudorapidity region $|\eta | < 2.2$. The distance of closest approach of each muon to the event vertex in the transverse plane must be less than 0.5$\,\text {cm}$ and a fit of the two muons to a common vertex must have a $\chi ^2$ probability greater than 0.5%. The invariant mass of the dimuon system must lie within ±5 times the experimental mass resolution (typically about 35$\,\text {MeV}$) of the world-average $\mathrm {J}/\psi$ mass [2].

The offline selection starts from $\mathrm {J}/\psi$ candidates that are reconstructed from pairs of oppositely charged muons. The standard CMS muon reconstruction procedure [25] is used to identify the muons, which requires multiple hits in the pixel, strip, and muon detectors with a consistent trajectory throughout. The offline selection requirements on the dimuon system replicate the trigger selection. From the sample of collected $\mathrm {J}/\psi$ events, candidate b hadrons are reconstructed by combining a $\mathrm {J}/\psi$ candidate with track(s) or reconstructed neutral particles, depending on the decay mode. Only tracks that pass the standard CMS high-purity requirements [17] are used. The b hadron candidate is fitted to a common vertex with the appropriate masses assigned to the charged tracks and the dimuon invariant mass constrained to the world-average $\mathrm {J}/\psi$ mass [2]. In fits that include a $\mathrm {K^0_S}$ or $\mathrm {\Lambda }^0$ hadron, the world-average mass is used for those particles. Primary vertices (PV) are fitted from the reconstructed tracks using an estimate of the proton–proton interaction region (beamspot) as a constraint. The PV having the smallest pointing angle, defined as the angle between the reconstructed $\mathrm {b}$ hadron momentum and the vector joining the PV with the decay vertex, is used. As the proper decay times are measured in the transverse plane, where the PV position is dominated by the beamspot, the choice of PV has little effect on the analysis and is accounted for as a systematic uncertainty.

### Reconstruction of ${\mathrm {B}^{+}}$, ${\mathrm {B}^0}$, $\mathrm {B}^0_\mathrm {s}$, and $\varLambda _\mathrm {b}^0$ hadrons

The ${\mathrm {B}^{+}}$, ${\mathrm {B}^0}$, $\mathrm {B}^0_\mathrm {s}$, and $\varLambda _\mathrm {b}^0$ hadrons are reconstructed in the decays ${\mathrm {B}^{+}}\!\rightarrow \! \mathrm {J}/\psi \mathrm {K^+}$, ${\mathrm {B}^0}\!\rightarrow \! \mathrm {J}/\psi \mathrm {K^0_S}$, ${\mathrm {B}^0}\!\rightarrow \! \mathrm {J}/\psi \mathrm {K^{*}(892)}{}^{0}$, $\mathrm {B}^0_\mathrm {s} \!\rightarrow \! \mathrm {J}/\psi \mathrm {\pi ^+}\mathrm {\pi ^-}$, $\mathrm {B}^0_\mathrm {s} \!\rightarrow \! \mathrm {J}/\psi \mathrm {\phi (1020)}$, and $\varLambda _\mathrm {b}^0\!\rightarrow \!\mathrm {J}/\psi \mathrm {\Lambda }^{0}$. The $\mathrm {K^{*}(892)}{}^{0}$, $\mathrm {K^0_S}$, $\mathrm {\phi (1020)}$, and $\mathrm {\Lambda }^{0}$ candidates are reconstructed from pairs of oppositely charged tracks that are consistent with originating from a common vertex. Because of the lack of charged particle identification, the labelling of tracks as pions, kaons, and protons simply means the mass that is assigned to the track. The mass assignments for the $\mathrm {K^0_S}$ and $\mathrm {\phi (1020)}$ decay products are unambiguous (either both pions or both kaons). For the kinematic region considered in this analysis, simulations show that the proton always corresponds to the track with the larger momentum (leading track) from the $\mathrm {\Lambda }^{0}$ decay. The $\mathrm {K^{*}(892)}{}^{0}$ candidates are constructed from a pair of tracks with kaon and pion mass assignments.

Since two $\mathrm {K^{*}(892)}{}^{0}$ candidates can be formed with a single pair of tracks, we select the combination for which the mass of the $\mathrm {K^{*}(892)}{}^{0}$ candidate is closest to the world-average value [2]. This selects the correct combination 88% of the time.

All tracks must have a transverse momentum greater than 0.5$\,\text {GeV}$. The decay vertices of the $\mathrm {K^0_S}$ and $\mathrm {\Lambda }^{0}$ particles are required to have a transverse decay length larger than 15$\sigma$ and their two decay products must each have a transverse impact parameter of at least 2$\sigma$, where the distances are with respect to the beamspot and $\sigma$ is the calculated uncertainty in the relevant quantity. The intermediate candidate states $\mathrm {K^{*}(892)}{}^{0}$, $\mathrm {K^0_S}$, $\mathrm {\phi (1020)}$, and $\mathrm {\Lambda }^{0}$ are selected if they lie within the following mass regions that correspond to 1–2 times the experimental resolution or natural width around the nominal mass: $0.7960<M(\mathrm {K^+}\mathrm {\pi ^-})<0.9880$ $\,\text {GeV}$, $0.4876<M(\mathrm {\pi ^+}\mathrm {\pi ^-})<0.5076$ $\,\text {GeV}$, $1.0095< M(\mathrm {K^+}\mathrm {K^-}) < 1.0295$ $\,\text {GeV}$, and $1.1096<M(\mathrm {p}\mathrm {\pi ^-})<1.1216$ $\,\text {GeV}$. The accepted mass region of the $\mathrm {\pi ^+}\mathrm {\pi ^-}$ system in $\mathrm {B}^0_\mathrm {s} \!\rightarrow \! \mathrm {J}/\psi \mathrm {\pi ^+}\mathrm {\pi ^-}$ decay is $0.9240< M(\mathrm {\pi ^+}\mathrm {\pi ^-}) < 1.0204$ $\,\text {GeV}$. The $\mathrm {K^0_S}$ contamination in the $\mathrm {\Lambda }^{0}$ sample is removed by discarding candidates in which the leading particle in the $\mathrm {\Lambda }^{0}$ decay is assigned the pion mass and the resulting $\mathrm {\pi ^+}\mathrm {\pi ^-}$ invariant mass is in the range $0.4876<M(\mathrm {\pi ^+}\mathrm {\pi ^-})<0.5076$ $\,\text {GeV}$. Conversely, the $\mathrm {\Lambda }^{0}$ contamination is removed from the $\mathrm {K^0_S}$ sample by discarding candidates in the $\mathrm {p}\mathrm {\pi ^-}$ mass region $1.1096<M(\mathrm {p}\mathrm {\pi ^-})<1.1216$ $\,\text {GeV}$, when the proton mass is assigned to the leading pion from the $\mathrm {K^0_S}$ decay. The $p_{\mathrm {T}}$ of the $\mathrm {K^+}$ candidate track from the ${\mathrm {B}^{+}}$ decay must be larger than 1$\,\text {GeV}$. The $p_{\mathrm {T}}$ of the $\mathrm {\pi ^+}\mathrm {\pi ^-}$ system in $\mathrm {B}^0_\mathrm {s} \!\rightarrow \! \mathrm {J}/\psi \mathrm {\pi ^+}\mathrm {\pi ^-}$ decays and the $\mathrm {K^{*}(892)}{}^{0}$ candidates in ${\mathrm {B}^0}\!\rightarrow \! \mathrm {J}/\psi \mathrm {K^{*}(892)}{}^{0}$ decays must be greater than 3.5$\,\text {GeV}$, with the leading (trailing) charged hadrons in these decays required to have a $p_{\mathrm {T}}$ greater than 2.5 (1.5)$\,\text {GeV}$. The $p_{\mathrm {T}}$ of the $\mathrm {b}$ hadrons must be at least 13$\,\text {GeV}$, except for the $\mathrm {B}^0_\mathrm {s} \!\rightarrow \! \mathrm {J}/\psi \mathrm {\phi (1020)}$ decay where no requirement is imposed. The $p_{\mathrm {T}}$ of the leading track from the $\mathrm {K^0_S}$ and $\mathrm {\Lambda }^{0}$ decays must be larger than 1.8$\,\text {GeV}$. The minimum $p_{\mathrm {T}}$ for the kaons forming a $\mathrm {\phi (1020)}$ candidate is 0.7$\,\text {GeV}$.

The $\mathrm {b}$ hadron vertex $\chi ^2$ probability is required to be greater than 0.1% in the $\mathrm {B}^0_\mathrm {s} \!\rightarrow \! \mathrm {J}/\psi \mathrm {\phi (1020)}$ channel only. The lifetime measurement is limited to events in which the $\mathrm {b}$ hadron $ct$ is greater than 0.02$\,\text {cm}$ to avoid resolution and reconstruction effects present in the low-$ct$ region. No attempt is made to select a single b hadron candidate in the relatively rare $(<1\%)$ events in which more than one b hadron candidate is found.

### Reconstruction of $\mathrm {B}_\mathrm {c}^+ \!\rightarrow \!\mathrm {J}/\psi \mathrm {\pi ^+}$

The $\mathrm {B}_\mathrm {c}^+$ lifetime is measured using the method developed by the LHCb Collaboration [12] in which the measured difference in total widths between the $\mathrm {B}_\mathrm {c}^+$ and ${\mathrm {B}^{+}}$ mesons is used in combination with the precisely known ${\mathrm {B}^{+}}$ lifetime to obtain the $\mathrm {B}_\mathrm {c}^+$ lifetime. This method does not require modelling the background $ct$ distribution, avoiding a source of systematic uncertainty. The same reconstruction algorithm and selection criteria are used for both decays, $\mathrm {B}_\mathrm {c}^+ \!\rightarrow \!\mathrm {J}/\psi \mathrm {\pi ^+}$ and ${\mathrm {B}^{+}}\!\rightarrow \!\mathrm {J}/\psi \mathrm {K^+}$. As a result, the dependence of the efficiencies on the proper decay time is similar.

The charged hadron tracks are required to have at least 2 pixel hits, at least 6 tracker hits (strips and pixels together), a track fit $\chi ^2$ less than 3 times the number of degrees of freedom, and $|\eta |< 2.4$. The dimuon invariant mass is required to lie in the range ±3$\sigma$ from the nominal $\mathrm {J}/\psi$ meson mass, where $\sigma$ is the average resolution for the $\mathrm {J}/\psi$ signal, which depends on the $\mathrm {J}/\psi$ pseudorapidity and ranges from 35 to 50$\,\text {MeV}$. The $p_{\mathrm {T}}$ of the charged hadron tracks and the b hadrons are required to be greater than 3.3 and 10$\,\text {GeV}$, respectively. The b hadrons must have a rapidity of $|y|<2.2$, a vertex $\chi ^2$ probability greater than 5%, a dimuon vertex $\chi ^2$ probability greater than 1%, and $\cos \theta > 0.98$, where ${\cos \theta = \vec {L}_{xy}\cdot \vec {p}_{\mathrm {T},{\mathrm {B}}} / (|L_{xy}|\cdot |p_{\mathrm {T},{\mathrm {B}}}|)}$ and $\vec {L}_{xy}$ and $\vec {p}_{\mathrm {T},{\mathrm {B}}}$ refer to the transverse decay length and momentum of the ${\mathrm {B}^{+}}$ or $\mathrm {B}_\mathrm {c}^+$ mesons. The lifetime measurement is limited to events in which the b hadron has $ct > 0.01\,\text {cm}$, which ensures that the ratio of the $\mathrm {B}_\mathrm {c}^+$ to ${\mathrm {B}^{+}}$ meson efficiencies is constant versus $ct$. The analysis of the $\mathrm {B}_\mathrm {c}^+$ lifetime is described in Sect. 6.

## Measurement of the ${\mathrm {B}^0}$, $\mathrm {B}^0_\mathrm {s}$, and $\varLambda _\mathrm {b}^0$ lifetimes

For each decay channel, we perform a simultaneous fit to three input variables, the b hadron mass, $ct$, and $ct$ uncertainty ($\sigma _{ct}$). For the ${\mathrm {B}^{+}}$, ${\mathrm {B}^0}$, and $\varLambda _\mathrm {b}^0$ hadrons, an unbinned maximum-likelihood fit is performed with a probability density function (PDF) given by:

7 $$\begin{aligned} \mathrm {PDF}= & {} f_s\; M_{s}(M)\; T_{s}(ct)\; E_{s}(\sigma _{ct})\; \varepsilon (ct) \nonumber \\&+ (1-f_s)\; M_{b}(M)\; T_{b}(ct)\; E_{b}(\sigma _{ct}), \end{aligned}$$

where $f_s$ is the fraction of signal events, and $M_s$ ($M_b$), $T_s$ ($T_b$), and $E_s$ ($E_b$) are the functions describing the signal (background) distributions of the b hadron mass, $ct$, and $\sigma _{ct}$, respectively, while $\varepsilon$ is the efficiency function. These functions are derived below. For the $\mathrm {B}^0_\mathrm {s}$ modes, we use an extended maximum-likelihood fit in order to correctly incorporate background sources whose yields are obtained from the fit.

### Reconstruction and selection efficiency

The reconstruction and selection efficiency $\varepsilon$ for each decay mode is determined as a function of $ct$ by using fully simulated MC samples. This efficiency is defined as the generated $ct$ distribution of the selected events after reconstruction and selection divided by the $ct$ distribution obtained from an exponential decay with the lifetime set to the value used to generate the events. The efficiency for the $\mathrm {B}^0_\mathrm {s} \!\rightarrow \! \mathrm {J}/\psi \mathrm {\phi (1020)}$ channel is defined as the generated $ct$ distribution of the selected events after reconstruction divided by the sum of the two exponentials generated with the theoretical $\mathrm {B}^0_\mathrm {s} \!\rightarrow \! \mathrm {J}/\psi \mathrm {\phi (1020)}$ decay rate model [26]. In the theoretical model, the values of the physics parameters are set to those used in the simulated sample.

**Fig. 1:**
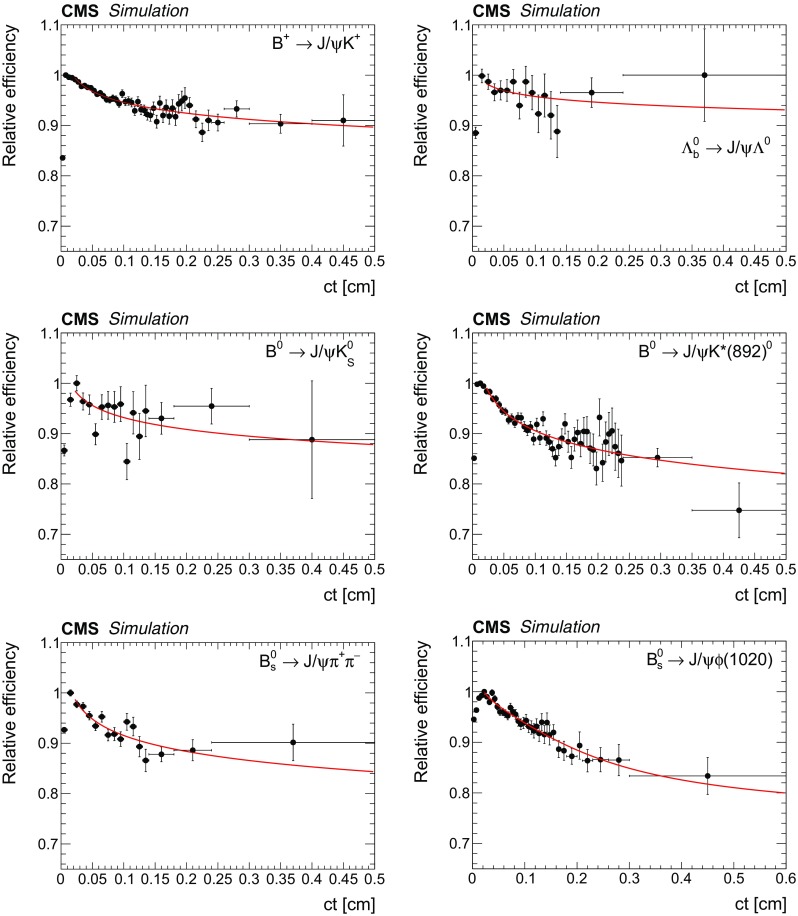
The combined reconstruction and selection efficiency from simulation versus $ct$ with a superimposed fit to an inverse power function for ${\mathrm {B}^{+}}\!\rightarrow \! \mathrm {J}/\psi \mathrm {K^+}$ (upper left), $\varLambda _\mathrm {b}^0\!\rightarrow \!\mathrm {J}/\psi \mathrm {\Lambda }^{0}$ (upper right), ${\mathrm {B}^0}\!\rightarrow \! \mathrm {J}/\psi \mathrm {K^0_S}$ (centre left), ${\mathrm {B}^0}\!\rightarrow \! \mathrm {J}/\psi \mathrm {K^{*}(892)}{}^{0}$ (centre right), $\mathrm {B}^0_\mathrm {s} \!\rightarrow \! \mathrm {J}/\psi \mathrm {\pi ^+}\mathrm {\pi ^-}$ (lower left), and $\mathrm {B}^0_\mathrm {s} \!\rightarrow \! \mathrm {J}/\psi \mathrm {\phi (1020)}$ (lower right). The efficiency scale is arbitrary

Figure 1 shows the efficiency as a function of $ct$ for the various decay modes, with an arbitrary normalization since only the relative efficiency is relevant. The efficiencies display a sharp rise as $ct$ increases from 0 to 0.01 $\text {cm}$, followed by a slow decrease as $ct$ increases further. The $ct$ efficiency is modelled with an inverse power function.

### Data modelling

Depending on the decay channel, the invariant mass distribution for the signal $M_s$ is modelled with one or two Gaussian functions, and a linear polynomial or an exponential function is used to model the combinatorial background $M_b$. For the $\mathrm {B}^0_\mathrm {s} \!\rightarrow \! \mathrm {J}/\psi \mathrm {\pi ^+}\mathrm {\pi ^-}$ decay, three additional terms are added to $M_b$ to include specific sources of background. The ${\mathrm {B}^0}\!\rightarrow \! \mathrm {J}/\psi \mathrm {\pi ^+}\mathrm {\pi ^-}$ decays are modelled by a Gaussian function, the ${\mathrm {B}^{+}}\!\rightarrow \! \mathrm {J}/\psi \mathrm {K^+}$ decays by a shape taken from simulation, and the ${\mathrm {B}^0}_{\mathrm {(d,s)}} \!\rightarrow \! \mathrm {J}/\psi \mathrm {h}^{+}_{1} \mathrm {h}^{-}_{2}$ decays, where $\mathrm {h}^{+}_{1}$ and $\mathrm {h}^{-}_{2}$ are charged hadron tracks that are not both pions, by a Gaussian function.

**Fig. 2:**
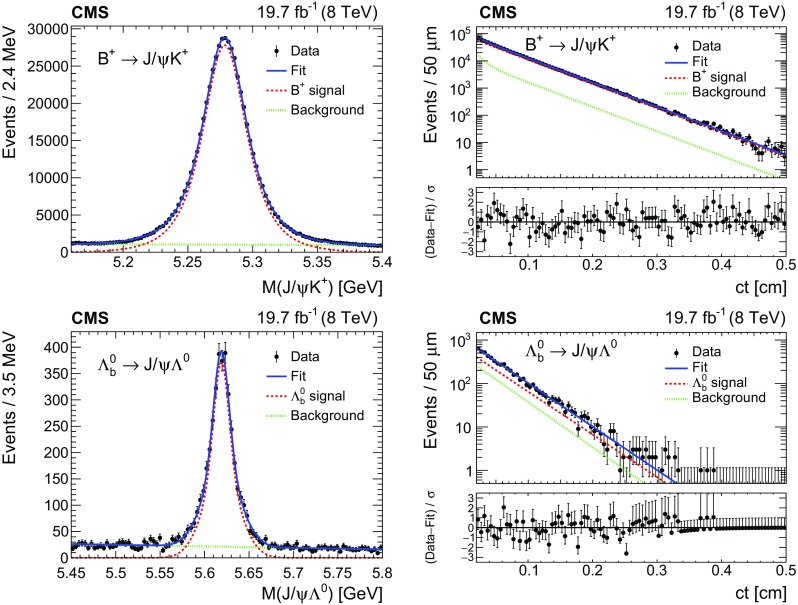
Invariant mass (left) and $ct$ (right) distributions for ${\mathrm {B}^{+}}$ (upper) and for $\varLambda _\mathrm {b}^0$ (lower) candidates. The curves are projections of the fit to the data, with the contributions from signal (dashed), background (dotted), and the sum of signal and background (solid) shown. the lower panels of the figures on the right show the difference between the observed data and the fit divided by the data uncertainty. The vertical bars on the data points represent the statistical uncertainties

**Fig. 3:**
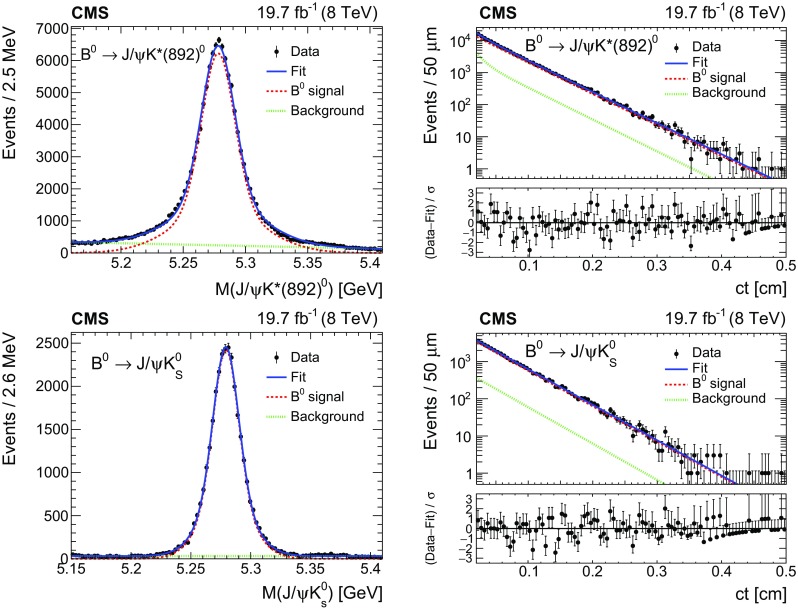
Invariant mass (left) and $ct$ (right) distributions for ${\mathrm {B}^0}$ candidates reconstructed from $\mathrm {J}/\psi \mathrm {K^{*}(892)}{}^{0}$ (upper) and $\mathrm {J}/\psi \mathrm {K^0_S}$ (lower) decays. The curves are projections of the fit to the data, with the contributions from signal (dashed), background (dotted), and the sum of signal and background (solid) shown. the lower panels of the figures on the right show the difference between the observed data and the fit divided by the data uncertainty. The vertical bars on the data points represent the statistical uncertainties

**Fig. 4:**
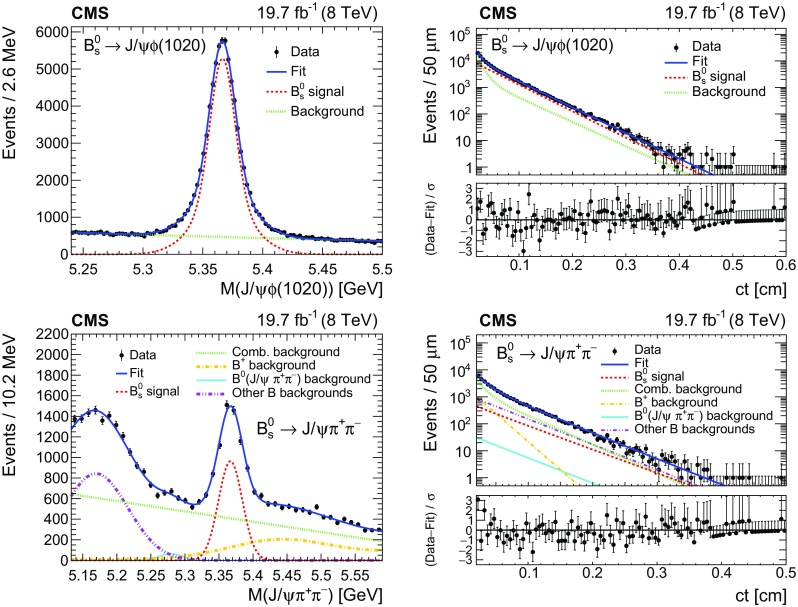
Invariant mass (left) and $ct$ (right) distributions for $\mathrm {B}^0_\mathrm {s}$ candidates reconstructed from $\mathrm {J}/\psi \mathrm {\phi (1020)}$ (upper) and $\mathrm {J}/\psi \mathrm {\pi ^+}\mathrm {\pi ^-}$ (lower) decays. The curves are projections of the fit to the data, with the full fit function (solid) and signal (dashed) shown for both decays, the total background (dotted) shown for the upper plots, and the combinatorial background (dotted), misidentified ${\mathrm {B}^{+}}\!\rightarrow \! \mathrm {J}/\psi \mathrm {K^+}$ background (dashed-dotted), ${\mathrm {B}^0}\!\rightarrow \! \mathrm {J}/\psi \mathrm {\pi ^+}\mathrm {\pi ^-}$ contribution (dashed-dotted-dotted-dotted), and partially reconstructed and other misidentified ${\mathrm {B}}$ backgrounds (dashed-dotted-dotted) shown for the lower plots. the lower panels of the figures on the right show the difference between the observed data and the fit divided by the data uncertainty. The vertical bars on the data points represent the statistical uncertainties

The signal $ct$ distribution $T_s$ is modelled by an exponential function convolved with the detector resolution and then multiplied by the function describing the reconstruction and selection efficiency. The resolution is described by a Gaussian function with the per-event width taken from the $ct$ uncertainty distribution. The backgrounds $T_b$ are described by a superposition of exponential functions convolved with the resolution. The number of exponentials needed to describe the background is determined from data events in the mass sideband regions for each decay mode.

The signal $E_s$ and background $E_b$ $\sigma _{ct}$ distributions are modelled with a sum of two gamma functions for the $\mathrm {B}^0_\mathrm {s} \!\rightarrow \! \mathrm {J}/\psi \mathrm {\phi (1020)}$ channel and two exponential functions convolved with a Gaussian function for the other channels. The background parameters are obtained from a fit to the mass sideband distributions. The signal parameters are obtained from a fit to the signal region after subtracting the background contribution using the mass sideband region to estimate the background. The parameters of the efficiency function and the functions modelling the $\sigma _{ct}$ distributions are kept constant in the fit. The remaining fit parameters are allowed to vary freely.

For the $\mathrm {B}^0_\mathrm {s} \!\rightarrow \! \mathrm {J}/\psi \mathrm {\pi ^+}\mathrm {\pi ^-}$ mode, the parameters of the mass model for the ${\mathrm {B}^{+}}\!\rightarrow \! \mathrm {J}/\psi \mathrm {K^+}$ contamination are taken from the simulation, and the yield and lifetime are determined by the fit. The mass of the ${\mathrm {B}^0}\!\rightarrow \! \mathrm {J}/\psi \mathrm {\pi ^+}\mathrm {\pi ^-}$ contamination is fixed to the weighted average of the masses measured from our two ${\mathrm {B}^0}$ decay modes, and the width of the Gaussian function is the same as the width used for the $\mathrm {B}^0_\mathrm {s} \!\rightarrow \! \mathrm {J}/\psi \mathrm {\pi ^+}\mathrm {\pi ^-}$ signal, corrected by a factor of $M_{{\mathrm {B}^0}}/M_{\mathrm {B}^0_\mathrm {s}}$. The lifetime of this contamination is fixed to the world-average value, corrected by the same factor as the width, and the yield is a free parameter of the fit.

### Fit results

The invariant mass and $$ct$$ distributions obtained from data are shown with the fit results superimposed in Figs. 2, 3 and 4. The $$ct$$ distributions are fitted in the range 0.02–0.50$$\,\text {cm}$$ for all modes except the $$\mathrm {B}^0_\mathrm {s} \!\rightarrow \! \mathrm {J}/\psi \mathrm {\phi (1020)}$$ channel, where the upper limit is increased to 0.60$$\,\text {cm}$$. The average lifetimes times the speed of light obtained from the fits are: $$c\tau _{{\mathrm {B}^{+}}} = 490.9 \pm 0.8\,\upmu \text {m} $$, $$c\tau _{{\mathrm {B}^0}\rightarrow \mathrm {J}/\psi \mathrm {K^{*}(892)}{}^{0}} = 453.0 \pm 1.6\,\upmu \text {m} $$, $$c\tau _{{\mathrm {B}^0}\rightarrow \mathrm {J}/\psi \mathrm {K^0_S}} = 457.8 \pm 2.7\,\upmu \text {m} $$, $$c\tau _{\mathrm {B}^0_\mathrm {s} \rightarrow \mathrm {J}/\psi \mathrm {\pi ^+}\mathrm {\pi ^-}} = 502.7 \pm 10.2\,\upmu \text {m} $$, $$c\tau _{\mathrm {B}^0_\mathrm {s} \rightarrow \mathrm {J}/\psi \mathrm {\phi (1020)}} = 445.2 \pm 2.0\,\upmu \text {m} $$, and $$c\tau _{\varLambda _\mathrm {b}^0} = 442.9 \pm 8.2\,\upmu \text {m} $$, where all uncertainties are statistical only. The $$\mathrm {B}^0_\mathrm {s} \!\rightarrow \! \mathrm {J}/\psi \mathrm {\phi (1020)}$$ value given here is uncorrected for two offsets described in Sect. 7. There is good agreement between the fitted functions and the data. The probabilities calculated from the $$\chi ^2$$ of the $$ct $$ distributions in Figs. 2, 3 and 4 all exceed 25%.

## Measurement of the $\mathrm {B}_\mathrm {c}^+$ lifetime

The decay time distribution for the signal $N_\mathrm {B}(ct)$ can be expressed as the product of an efficiency function $\varepsilon _\mathrm {B}(ct)$ and an exponential decay function $E_\mathrm {B}(ct) =\exp (-ct/c\tau _\mathrm {B})$, convolved with the time resolution function of the detector *r*(*ct*). The ratio of $\mathrm {B}_\mathrm {c}^+$ to ${\mathrm {B}^{+}}$ events at a given proper time can be expressed as

8 $$\begin{aligned} \frac{N_{\mathrm {B}_\mathrm {c}^+}(ct)}{N_{{\mathrm {B}^{+}}}(ct)} \equiv R(ct) = \frac{\varepsilon _{\mathrm {B}_\mathrm {c}^+}(ct) [r(ct)\otimes E_{\mathrm {B}_\mathrm {c}^+}(ct)]}{\varepsilon _{{\mathrm {B}^{+}}}(ct) [r(ct)\otimes E_{{\mathrm {B}^{+}}}(ct)]}. \end{aligned}$$

We have verified through studies of simulated pseudo-events that Eq. (8) is not significantly affected by the time resolution, and therefore this equation can be simplified to

9 $$\begin{aligned} R(ct) \approx R_{\varepsilon }(ct) \exp (-\varDelta \varGamma t), \end{aligned}$$

where the small effect from the time resolution is evaluated from MC simulations and is included in $R_{\varepsilon }(ct)$, which denotes the ratio of the $\mathrm {B}_\mathrm {c}^+$ and ${\mathrm {B}^{+}}$ efficiency functions. The quantity $\varDelta \varGamma$ is defined as

10 $$\begin{aligned} \varDelta \varGamma \equiv \varGamma _{\mathrm {B}_\mathrm {c}^+} - \varGamma _{{\mathrm {B}^{+}}} = \frac{1}{\tau _{\mathrm {B}_\mathrm {c}^+}} - \frac{1}{\tau _{{\mathrm {B}^{+}}}}. \end{aligned}$$

**Fig. 5:**
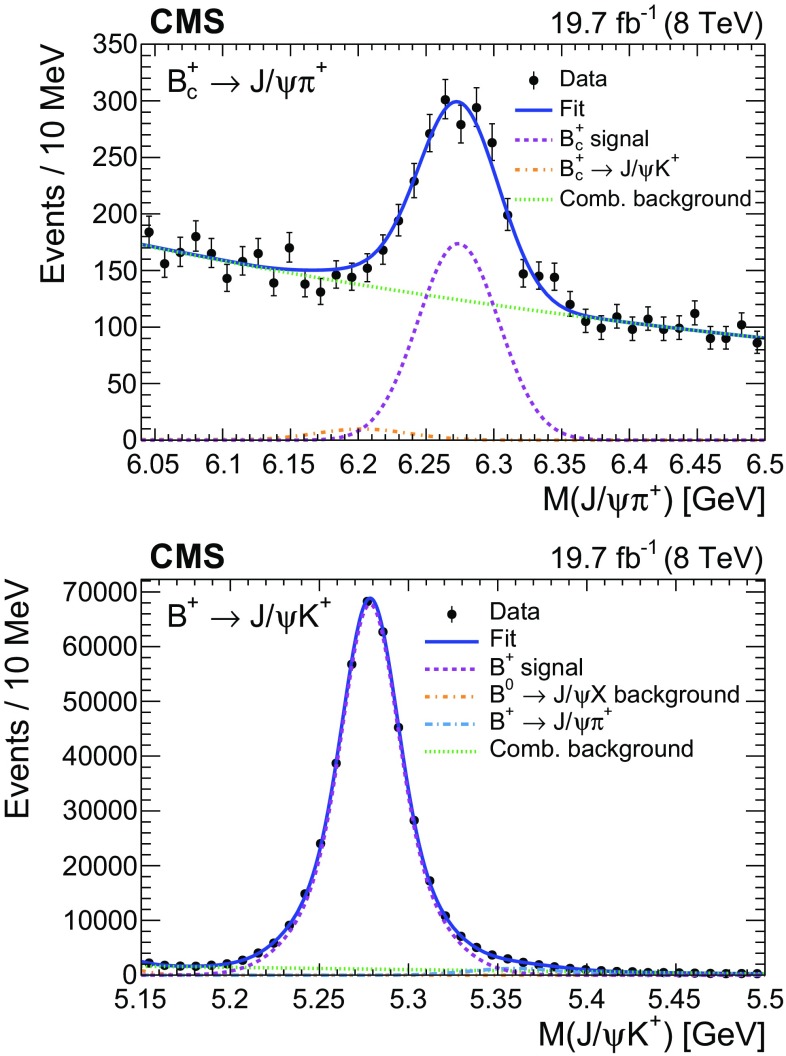
The $\mathrm {J}/\psi \mathrm {\pi ^+}$ invariant mass distribution (left) with the solid line representing the total fit, the dashed line the signal component, the dotted line the combinatorial background, and the dashed-dotted line the contribution from $\mathrm {B}_\mathrm {c}^+ \!\rightarrow \!\mathrm {J}/\psi \mathrm {K^+}$ decays. The $\mathrm {J}/\psi \mathrm {K^+}$ invariant mass distribution (right) with the solid line representing the total fit, the dashed line the signal component, the dotted-dashed curves the ${\mathrm {B}^{+}}\!\rightarrow \! {\mathrm {J}/\psi } \mathrm {\pi ^+}$ and ${\mathrm {B}^0}$ contributions, and the dotted curve the combinatorial background. The vertical bars on the data points represent the statistical uncertainties

The $\mathrm {B}_\mathrm {c}^+ \!\rightarrow \!\mathrm {J}/\psi \mathrm {\pi ^+}$ and ${\mathrm {B}^{+}}\!\rightarrow \!\mathrm {J}/\psi \mathrm {K^+}$ invariant mass distributions, shown in Fig. 5, are each fit with an unbinned maximum-likelihood estimator. The $\mathrm {J}/\psi \mathrm {\pi ^+}$ invariant mass distribution is fitted with a Gaussian function for the $\mathrm {B}_\mathrm {c}^+$ signal and an exponential function for the background. An additional background contribution from $\mathrm {B}_\mathrm {c}^+ \!\rightarrow \!\mathrm {J}/\psi \mathrm {K^+}$ decays is modelled from a simulated sample of $\mathrm {B}_\mathrm {c}^+ \!\rightarrow \!\mathrm {J}/\psi \mathrm {K^+}$ events, and its contribution is constrained using the value of the branching fraction relative to $\mathrm {J}/\psi \mathrm {\pi ^+}$  [27]. The $\mathrm {B}_\mathrm {c}^+ \!\rightarrow \!\mathrm {J}/\psi \mathrm {\pi ^+}$ signal yield is $1128 \pm 60$ events, where the uncertainty is statistical only. The ${\mathrm {B}^{+}}$ meson invariant mass distribution is fit with a sum of two Gaussian functions with a common mean for the signal and a second-order Chebyshev polynomial for the background. Additional contributions from partially reconstructed ${\mathrm {B}^0}$ and ${\mathrm {B}^{+}}$ meson decays are parametrized with functions determined from ${\mathrm {B}^{+}}\!\rightarrow \!\mathrm {J}/\psi \pi ^+$ and inclusive ${\mathrm {B}^0}\!\rightarrow \!\mathrm {J}/\psi \mathrm {X}$ MC samples.

### The fit model and results

**Fig. 6:**
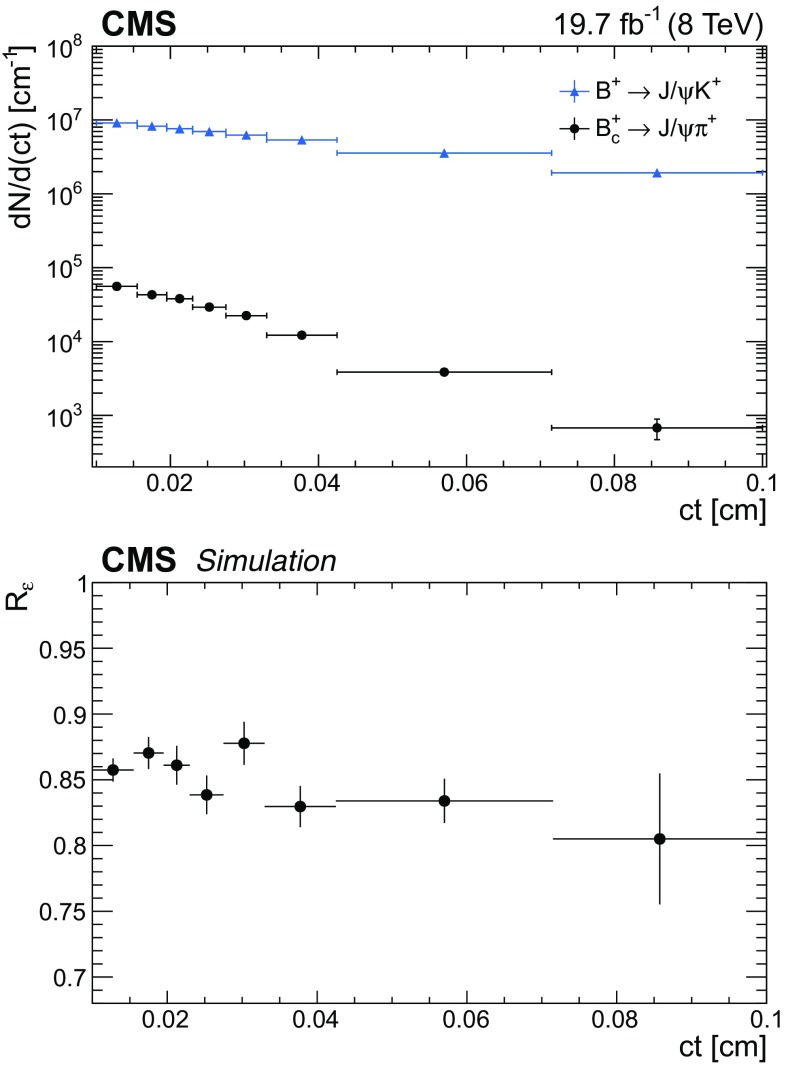
Yields of $\mathrm {B}_\mathrm {c}^+ \!\rightarrow \!\mathrm {J}/\psi \mathrm {\pi ^+}$ and ${\mathrm {B}^{+}}\!\rightarrow \!\mathrm {J}/\psi \mathrm {K^+}$ events (left) as a function of $ct$, normalized to the bin width, as determined from fits to the invariant mass distributions. Ratio of the $\mathrm {B}_\mathrm {c}^+$ and ${\mathrm {B}^{+}}$ efficiency distributions (right) as a function of $ct$, as determined from simulated events. The vertical bars on the data points represent the statistical uncertainties, and the horizontal bars show the bin widths

The $\mathrm {B}_\mathrm {c}^+$ lifetime is extracted through a binned $\chi ^2$ fit to the ratio of the efficiency-corrected $ct$ distributions of the $\mathrm {B}_\mathrm {c}^+ \!\rightarrow \!\mathrm {J}/\psi \mathrm {\pi ^+}$ and ${\mathrm {B}^{+}}\!\rightarrow \!\mathrm {J}/\psi \mathrm {K^+}$ channels. The $\mathrm {B}_\mathrm {c}^+$ and ${\mathrm {B}^{+}}$ $ct$ signal distributions from data are obtained by dividing the data sample into $ct$ bins and performing an unbinned maximum-likelihood fit to the $\mathrm {J}/\psi \mathrm {\pi ^+}$ and $\mathrm {J}/\psi \mathrm {K^+}$ invariant mass distribution in each bin, in the same manner as the fit to the full samples, except that the peak position and resolution are fixed to the values obtained by the fits to the full samples. Varied $ct$ bin widths are used to ensure a similar statistical uncertainty in the $\mathrm {B}_\mathrm {c}^+$ signal yield among the bins. The bin edges are defined by requiring a relative statistical uncertainty of 12% or better in each bin. The same binning is used for the ${\mathrm {B}^{+}}$ $ct$ distribution. The $\mathrm {B}_\mathrm {c}^+$ and ${\mathrm {B}^{+}}$ meson yields are shown versus $ct$ in the left plot of Fig. 6, where the number of signal events is normalized by the bin width. Efficiencies are obtained from the MC samples and are defined as the $ct$ distribution of the selected events after reconstruction divided by the $ct$ distribution obtained from an exponential decay with the lifetime set to the same value used to generate each MC sample. The ratio of the two efficiency distributions, using the same binning scheme as for the data, is shown in the right plot of Fig. 6.

**Fig. 7:**
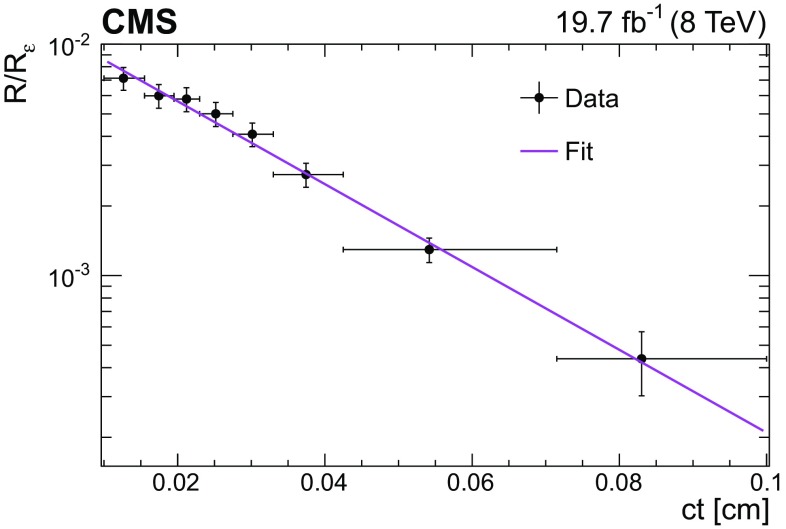
Ratio of the $\mathrm {B}_\mathrm {c}^+$ to ${\mathrm {B}^{+}}$ efficiency-corrected $ct$ distributions, $R/R_{\varepsilon }$, with a line showing the result of the fit to an exponential function. The vertical bars give the statistical uncertainty in the data, and the horizontal bars show the bin widths

The ratio of the $\mathrm {B}_\mathrm {c}^+$ to ${\mathrm {B}^{+}}$ efficiency-corrected $ct$ distributions, $R/R_{\varepsilon }$, is shown in Fig. 7, along with the result of a fit to an exponential function. The fit returns $\varDelta \varGamma = 1.24 \pm 0.09$ ps$^{-1}$. Using the known lifetime of the ${\mathrm {B}^{+}}$ meson, $c\tau _{{\mathrm {B}^{+}}}$ = 491.1 ± 1.2 $\,\upmu \text {m}$  [5], a measurement of the $\mathrm {B}_\mathrm {c}^+$ meson lifetime, $c\tau _{\mathrm {B}_\mathrm {c}^+}$ = 162.3 ± 7.8 $\,\upmu \text {m}$, is extracted, where the uncertainty is statistical only.

**Table 1:** Summary of the sources and values of systematic uncertainties in the lifetime measurements (in $\,\upmu \text {m}$). The total systematic uncertainty is the sum in quadrature of the individual uncertainties

Source	${\mathrm {B}^0}\!\!\rightarrow \! \mathrm {J}/\psi \mathrm {K^{*}(892)}{}^{0}$	${\mathrm {B}^0}\!\!\rightarrow \! \mathrm {J}/\psi \mathrm {K^0_S}$	$\mathrm {B}^0_\mathrm {s} \!\!\rightarrow \! \mathrm {J}/\psi \pi ^+\pi ^-$	$\mathrm {B}^0_\mathrm {s} \!\!\rightarrow \! \mathrm {J}/\psi \phi$	$\varLambda _\mathrm {b}^0\!\!\rightarrow \! \mathrm {J}/\psi {} \varLambda ^{\!0}$
MC statistical uncertainty	1.1	2.4	2.0	0.6	2.3
Mass modelling	0.3	0.4	0.2	0.4	0.9
$ct$ modelling	0.1	0.1	0.4	0.0	0.1
${\mathrm {B}^{+}}$ contamination	–	–	1.4	–	–
Mass window of $\pi ^+ \pi ^-$	–	–	1.8	–	–
$\mathrm {K}^\pm \pi ^\mp$ mass assignment	0.3	–	–	–	–
$ct$ range	–	–	–	0.1	–
S-wave contamination	–	–	–	0.4	–
Absolute *ct* accuracy	1.3	1.3	1.4	1.3	1.3
Total ($\upmu \hbox {m}$)	1.8	2.8	3.4	1.5	2.8

## Systematic uncertainties

The systematic uncertainties can be divided into uncertainties common to all the measurements, and uncertainties specific to a decay channel. Table 1 summarizes the systematic uncertainties for the sources considered below and the total systematic uncertainty in the $\mathrm {B}^0_\mathrm {s}$, ${\mathrm {B}^0}$, and $\varLambda _\mathrm {b}^0$ lifetime measurements. The systematic uncertainties in $\varDelta \varGamma$ and the $\mathrm {B}_\mathrm {c}^+$ meson lifetime are collected in Table 2. Using the known lifetime of the ${\mathrm {B}^{+}}$ meson, the uncertainties in $\varDelta \varGamma$ are converted into uncertainties in the $\mathrm {B}_\mathrm {c}^+$ meson lifetime measurement. The uncertainty in the $\mathrm {B}_\mathrm {c}^+$ meson lifetime due to the uncertainty in the ${\mathrm {B}^{+}}$ meson lifetime [5] is quoted separately.

We have verified that the results are stable against changes in the selection requirements on the quality of the tracks and vertices, the kinematic variables, and $ct$, as well as in detector regions and data-taking periods. The effect of replacing the mass of the b hadron in the $ct$ definition of Eq. (1) from the world-average to the reconstructed candidate mass is found to be negligible. The lifetimes for all decay channels were measured by treating MC samples as data. No bias was found and all results were consistent with the input lifetimes of the generated samples.

**Table 2:** Summary of the systematic uncertainties in the $\varDelta \varGamma$ and $c\tau _{\mathrm {B}_\mathrm {c}^+}$ measurements

Source	$\varDelta \varGamma$ (ps$^{-1}$)	$c\tau _{\mathrm {B}_\mathrm {c}^+}$ ($\upmu \text {m}$)
MC statistical uncertainty	0.01	1.2
Mass modelling	0.04	3.4
PV selection	0.02	2.0
Detector alignment	0.01	0.6
Total uncertainty	0.05	4.2

### Common systematic uncertainties

1. Statistical uncertainty in the MC samples
  The number of events in the simulation directly affects the accuracy of the efficiency determination. In the case of the $\mathrm {B}^0_\mathrm {s}$, ${\mathrm {B}^0}$, and $\varLambda _\mathrm {b}^0$ lifetime measurements, 1000 efficiency curves are generated with variations of the parameter values. The parameter values are sampled using a multivariate Gaussian PDF that is constructed from the covariance matrix of the efficiency fit. The analysis is performed 1000 times, varying the parameters of the efficiency function. The distribution of the measured lifetimes is fitted with a Gaussian function, whose width is taken as the systematic uncertainty associated with the finite size of the simulated samples. In the measurement of the $\mathrm {B}_\mathrm {c}^+$ lifetime, the bin-by-bin statistical uncertainty in the efficiency determination is propagated to the *R*(*ct*) distribution, the fit is performed, and the difference in quadrature of the uncertainty in $\varDelta \varGamma$ with respect to the nominal value is taken as the systematic uncertainty.
2. Modelling of the mass distribution shape
  Biases related to the modelling of the shapes of the b hadron mass signal and background PDFs are quantified by changing the signal and background PDFs individually and using the new models to fit the data. For the ${\mathrm {B}^0}$, $\mathrm {B}^0_\mathrm {s}$, and $\mathrm {\Lambda }_\mathrm {b}^0$ lifetime measurements, the background model is changed to a higher-degree polynomial, a Chebyshev polynomial, or an exponential function, and the signal model is changed from two Gaussian functions to a single Gaussian function or a sum of three Gaussian functions. Differences in the measured lifetime between the results of the nominal and alternative models are used to estimate the systematic uncertainty, with the variations due to the modelling of signal and background components evaluated separately and added in quadrature. For the $\mathrm {B}_\mathrm {c}^+$ lifetime measurement, the signal peak is alternatively modelled with a Crystal Ball distribution [28]. The alternative description for the background is a first-order Chebyshev distribution. The removal of the Cabibbo-suppressed $\mathrm {B}_\mathrm {c}^+ \!\rightarrow \!\mathrm {J}/\psi \mathrm {K^+}$ contribution is also considered. The maximum deviation of the signal yield in each $ct$ bin from the nominal value is propagated to the statistical uncertainty in the per-bin yield. The fit to *R*(*ct*) is performed and the difference in quadrature between the uncertainty from this fit and the nominal measurement is taken as the systematic uncertainty.

### Channel-specific systematic uncertainties

1. Modelling of the background $ct$ shape in the $\mathrm {B}^0_\mathrm {s}$, ${\mathrm {B}^0}$, and $\varLambda _\mathrm {b}^0$ channels
  To estimate a systematic uncertainty due to the $ct$ background model, we add an additional background contribution modelled with its own lifetime, and compare the result to that obtained with the nominal fit model. The difference between the results of the nominal and alternative fit models is used as the systematic uncertainty from the $ct$ shape modelling.
2. The ${\mathrm {B}^{+}}$ contamination in the $\mathrm {B}^0_\mathrm {s} \!\rightarrow \! \mathrm {J}/\psi \mathrm {\pi ^+}\mathrm {\pi ^-}$ sample
  In the nominal fit, the yield and lifetime of the ${\mathrm {B}^{+}}\!\rightarrow \!\mathrm {J}/\psi \mathrm {K^+}$ contamination are determined from the fit with the mass shape obtained from simulation. An alternative estimate of the $\mathrm {J}/\psi \mathrm {K^+}$ contamination is obtained from data by taking the leading pion of the $\mathrm {B}^0_\mathrm {s} \!\rightarrow \! \mathrm {J}/\psi \mathrm {\pi ^+}\mathrm {\pi ^-}$ decay to be the kaon. The lifetime and yield of the ${\mathrm {B}^{+}}\!\rightarrow \!\mathrm {J}/\psi \mathrm {K^+}$ decays contaminating the $\mathrm {B}^0_\mathrm {s} \!\rightarrow \! \mathrm {J}/\psi \mathrm {\pi ^+}\mathrm {\pi ^-}$ sample are determined from a fit of the ${\mathrm {B}^{+}}$ signal candidates in the $\mathrm {B}^0_\mathrm {s} \!\rightarrow \! \mathrm {J}/\psi \mathrm {\pi ^+}\mathrm {\pi ^-}$ sample, with the mass shape also obtained from the data. The difference between the $\mathrm {B}^0_\mathrm {s}$ lifetime found with this model and the nominal model is considered as the systematic uncertainty due to ${\mathrm {B}^{+}}$ contamination.
3. Invariant mass window of the $\pi ^+ \pi ^-$ in the $\mathrm {B}^0_\mathrm {s} \!\rightarrow \! \mathrm {J}/\psi \mathrm {\pi ^+}\mathrm {\pi ^-}$ channel
  Although the events selected by the $\pi ^+\pi ^-$ mass window are dominated by the $f_0(980)$, its width is not well known and possible backgrounds under the $f_0(980)$ peak could be increased or decreased, depending on the mass window. The effect on the lifetime is studied by using mass windows of $\pm 30$ and $\pm 80$ $\,\text {MeV}$ around the signal peak, compared to the nominal fit result with a ±50$\,\text {MeV}$ window. The maximum variation of the lifetime is taken as the systematic uncertainty.
4. The $\mathrm {K^+}\mathrm {\pi ^-}$ mass assignments for $\mathrm {K^{*}(892)}{}^{0}$ candidates in the ${\mathrm {B}^0}\!\rightarrow \! \mathrm {J}/\psi \mathrm {K^{*}(892)}{}^{0}$ channel
  The $\mathrm {K^{*}(892)}{}^{0}$ candidates are constructed from a pair of tracks with kaon and pion mass assignments. The combination with invariant mass closest to the world-average $\mathrm {K^{*}(892)}{}^{0}$ mass is chosen to reconstruct the ${\mathrm {B}^0}$ candidate. To estimate the effect on the lifetime due to a possible misassignment of kaon and pion, both combinations are discarded if both are within the natural width of the $\mathrm {K^{*}(892)}{}^{0}$ mass, and the difference between the lifetime obtained with this sample and the nominal sample is taken as the systematic uncertainty.
5. The $ct$ range in the $\mathrm {B}^0_\mathrm {s} \!\rightarrow \! \mathrm {J}/\psi \mathrm {\phi (1020)}$ channel
  Since the $ct>0.02$ cm requirement distorts the fractions of heavy and light mass eigenstates, the measured $\mathrm {B}^0_\mathrm {s}$ effective lifetime must be corrected. The correction and systematic uncertainty are quantified analytically. The correction to the effective lifetime is
  11 $$\begin{aligned} \delta _{ct}= & {} c\tau _{\text {eff}}^{\mathrm {cut}} - c\tau _{\text {eff}} \nonumber \\= & {} \frac{ (1-|A_\perp |^2) (c\tau _{\mathrm {L}})^2 \mathrm {e}^{-a/c\tau _\mathrm {L}} + |A_\perp |^2 (c\tau _{\mathrm {H}})^2 \mathrm {e}^{-a/c\tau _\mathrm {H}} }{(1-|A_\perp |^2) c\tau _{\mathrm {L}} \mathrm {e}^{-a/c\tau _\mathrm {L}} + |A_\perp |^2 c\tau _{\mathrm {H}} \mathrm {e}^{-a/c\tau _\mathrm {H}} } \nonumber \\&- \frac{ (1-|A_\perp |^2) (c\tau _{\mathrm {L}})^2 + |A_\perp |^2 (c\tau _{\mathrm {H}})^2 }{ (1-|A_\perp |^2) c\tau _{\mathrm {L}}+ |A_\perp |^2 c\tau _{\mathrm {H}} } , \end{aligned}$$
  where the first term represents the effective lifetime in the presence of a $ct>a$ requirement and the latter term is the unbiased effective lifetime. In this analysis, *a* is equal to 0.02$\,\text {cm}$. The world-average values [2] for $c\tau _\mathrm {H}=482.7 \pm 3.6\,\upmu \text {m}$, $c\tau _\mathrm {L}= 426.3 \pm 2.4\,\upmu \text {m}$, and ${|A_\perp |^2 = 0.250 \pm 0.006}$ are used to obtain the correction ${\delta _{ct} = 0.62 \pm 0.10\,\upmu \text {m}}$.
6. The S-wave contamination in the $\mathrm {B}^0_\mathrm {s} \!\rightarrow \! \mathrm {J}/\psi \mathrm {\phi (1020)}$ channel
  The $\mathrm {B}^0_\mathrm {s}$ candidates reconstructed in the $\mathrm {J}/\psi \mathrm {\phi (1020)}$ final state contain a small fraction of nonresonant and CP-odd $\mathrm {B}^0_\mathrm {s} \!\rightarrow \! \mathrm {J}/\psi \mathrm {K^+}\mathrm {K^-}$ decays, where the invariant mass of the two kaons happens to be near the $\phi$ meson mass. The fraction of $\mathrm {B}^0_\mathrm {s} \!\rightarrow \! \mathrm {J}/\psi \mathrm {K^+}\mathrm {K^-}$ decays among the selected events is measured in the weak mixing phase analysis [9] to be $f_\mathrm {S} = (1.2^{+0.9}_{-0.7})$%. Because of the different trigger and signal selection criteria of the present analysis, the S-wave fraction is corrected according to the simulation to be $(1.5^{+1.1}_{-0.9})$%. The bias caused by the contamination of nonresonant $\mathrm {B}^0_\mathrm {s} \!\rightarrow \! \mathrm {J}/\psi \mathrm {K^+}\mathrm {K^-}$ decays is estimated by generating two sets of pseudo-experiments, one with just $\mathrm {B}^0_\mathrm {s} \!\rightarrow \! \mathrm {J}/\psi \mathrm {\phi (1020)}$ events and one with a fraction of S-wave events based on the measured S-wave fraction and its uncertainty. The difference in the average of the measured lifetimes of these two samples is 0.74$\,\upmu \text {m}$, which is used to correct the measured lifetime. The systematic uncertainty associated with this correction is obtained by taking the difference in quadrature between the standard deviation of the distribution of lifetime results from the pseudo-experiments with and without the S-wave contribution.
7. PV selection in the $\mathrm {B}_\mathrm {c}^+ \!\rightarrow \!\mathrm {J}/\psi \mathrm {\pi ^+}$ channel
  From the multiple reconstructed PVs in an event, one is selected to compute the $ct$ value of the candidate. Two alternative methods to select the PV position are studied: using the centre of the beamspot and selecting the PV with the largest sum of track $p_{\mathrm {T}}$. While all three methods are found to be effective and unbiased, there were small differences, and the maximum deviation with respect to the nominal choice is taken as the systematic uncertainty. The ${\mathrm {B}^{+}}$ and $\mathrm {B}_\mathrm {c}^+$ primary vertex choices were changed coherently.
8. Detector alignment in the $\mathrm {B}_\mathrm {c}^+ \!\rightarrow \!\mathrm {J}/\psi \mathrm {\pi ^+}$ channel
  Possible effects on the lifetime due to uncertainties in the detector alignment [29] are investigated for each decay topology using 20 different simulated samples with distorted geometries. These distortions include expansions in the radial and longitudinal dimensions, rotations, twists, offsets, etc. The amount of misalignment is chosen such that it is large enough to be detected and corrected by the alignment procedure. The standard deviation of the lifetimes for the tested scenarios is taken as the systematic uncertainty from this source. The ${\mathrm {B}^{+}}$ and $\mathrm {B}_\mathrm {c}^+$ geometries were changed coherently.
9. Absolute *ct* accuracy in the $\mathrm {B}^0_\mathrm {s}$, ${\mathrm {B}^0}$, and $\varLambda _\mathrm {b}^0$ lifetime measurements
  The lifetime of the most statistically precise mode (${\mathrm {B}^{+}}\!\rightarrow \! \mathrm {J}/\psi \mathrm {K^+}$) is used to validate the accuracy of the simulation and various detector calibrations. The difference between our measurement of $490.9 \pm 0.8\,\upmu \text {m}$ (statistical uncertainty only) and the world-average value of $491.1 \pm 1.2\,\upmu \text {m}$ [5] is $0.2 \pm 1.4\,\upmu \text {m}$. This implies a limit to the validation of $1.4/491 = 0.3\%$. Four systematic effects that we expect to be included were checked independently. The systematic uncertainties from PV selection and detector alignment were found to be 0.7$\,\upmu \text {m}$ and 0.3–0.7$\,\upmu \text {m}$, respectively. Varying the efficiency functional form changed the lifetimes by 0.3–0.6$\,\upmu \text {m}$, while varying $\sigma _{ct}$ by factors of 0.5 and 2.0 resulted in lifetime differences of no more than $0.2\,\upmu \text {m}$. As the sum in quadrature of these uncertainties is less than that obtained from the ${\mathrm {B}^{+}}$ lifetime comparison, we assign a value of 0.3% as the systematic uncertainty for the absolute *ct* accuracy.

## Lifetime measurement results

Our final results for the ${\mathrm {B}^0}$, $\mathrm {B}^0_\mathrm {s}$, and $\varLambda _\mathrm {b}^0$ hadron lifetimes are:

12 $$\begin{aligned}&c\tau _{{\mathrm {B}^0}\rightarrow \mathrm {J}/\psi \mathrm {K^{*}(892)}{}^{0}} = 453.0 \pm 1.6\,\text {(stat)} \pm 1.8\,\text {(syst)} \,\upmu \text {m}, \end{aligned}$$

13 $$\begin{aligned}&c\tau _{{\mathrm {B}^0}\rightarrow \mathrm {J}/\psi \mathrm {K^0_S}} = 457.8 \pm 2.7\,\text {(stat)} \pm 2.8\,\text {(syst)} \,\upmu \text {m},\end{aligned}$$

14 $$\begin{aligned}&c\tau _{\mathrm {B}^0_\mathrm {s} \rightarrow \mathrm {J}/\psi \mathrm {\pi ^+}\mathrm {\pi ^-}} = 502.7 \pm 10.2\,\text {(stat)} \pm 3.4\,\text {(syst)} \,\upmu \text {m}, \end{aligned}$$

15 $$\begin{aligned}&c\tau _{\mathrm {B}^0_\mathrm {s} \rightarrow \mathrm {J}/\psi \mathrm {\phi (1020)}} = 443.9 \pm 2.0\,\text {(stat)} \pm 1.5\,\text {(syst)} \,\upmu \text {m},\end{aligned}$$

16 $$\begin{aligned}&c\tau _{\varLambda _\mathrm {b}^0{}} = 442.9 \pm 8.2\,\text {(stat)} \pm 2.8\,\text {(syst)} \,\upmu \text {m}. \end{aligned}$$

The value of the $\mathrm {B}^0_\mathrm {s}$ lifetime using the $\mathrm {J}/\psi \mathrm {\phi (1020)}$ decay has been corrected for the $ct$ range and S-wave contamination effects described in Sect. 7. The lifetime ratios $\tau _{\mathrm {B}^0_\mathrm {s}}/\tau _{{\mathrm {B}^0}}$ and $\tau _{\varLambda _\mathrm {b}^0}/\tau _{{\mathrm {B}^0}}$ have been determined using the decay channels ${\mathrm {B}^0}\!\rightarrow \! \mathrm {J}/\psi \mathrm {K^{*}(892)}{}^{0}$, $\mathrm {B}^0_\mathrm {s} \!\rightarrow \! \mathrm {J}/\psi \mathrm {\phi (1020)}$, and $\varLambda _\mathrm {b}^0\!\rightarrow \!\mathrm {J}/\psi \mathrm {\Lambda }^{0}$. Including the statistical and correlated and uncorrelated systematic uncertainties, the results are:

17 $$\begin{aligned}&\tau _{\varLambda _\mathrm {b}^0}/\tau _{{\mathrm {B}^0}\rightarrow \mathrm {J}/\psi \mathrm {K^{*}(892)}{}^{0}} \nonumber \\&\quad = 0.978 \pm 0.018\,\text {(stat)} \pm 0.006\,\text {(syst)}, \end{aligned}$$

18 $$\begin{aligned}&\tau _{\varLambda _\mathrm {b}^0}/\tau _{{\mathrm {B}^0}\rightarrow \mathrm {J}/\psi \mathrm {K^{*}(892)}{}^{0}} \nonumber \\&\quad = 0.978 \pm 0.018\,\text {(stat)} \pm 0.006\,\text {(syst)}, \end{aligned}$$

These ratios are compatible with the current world-average values.

The measured lifetimes for the ${\mathrm {B}^0}$ meson in the two different channels are in agreement. Combining the two results, including the statistical and the correlated and uncorrelated systematic uncertainties, gives $c\tau _{{\mathrm {B}^0}} = 454.1 \pm 1.4\,\text {(stat)} \pm 1.7\,\text {(syst)} \,\upmu \text {m}$. The lifetime measurements can also be used to estimate $\varGamma _\mathrm {d}$ and $\varDelta \varGamma _\mathrm {d}$ [6]. In the standard model, the effective lifetimes of the two ${\mathrm {B}^0}$ decay modes can be written as:

19 $$\begin{aligned} \tau _{{\mathrm {B}^0}\rightarrow \mathrm {J}/\psi \mathrm {K^{*}(892)}{}^{0}}&= \frac{1}{\varGamma _\mathrm {d}} \left( \frac{1}{1-y_\mathrm {d}^2}\right) \left( \frac{1+2\cos {(2\beta )}y_\mathrm {d}+y_\mathrm {d}^2}{1+\cos {(2\beta )}y_\mathrm {d}}\right) , \end{aligned}$$

20 $$\begin{aligned} \tau _{{\mathrm {B}^0}\rightarrow \mathrm {J}/\psi \mathrm {K^0_S}}&= \frac{1}{\varGamma _\mathrm {d}} \left( \frac{1+y_\mathrm {d}^2}{1-y_\mathrm {d}^2}\right) , \end{aligned}$$

where $y_\mathrm {d}=\varDelta \varGamma _\mathrm {d}/2\varGamma _\mathrm {d}$, and $\beta =(21.9\pm 0.7)^\circ$ [5] is one of the CKM unitarity triangle angles. Using our measured values for the two ${\mathrm {B}^0}$ lifetimes, we fit for $\varGamma _\mathrm {d}$ and $\varDelta \varGamma _\mathrm {d}$ and use the values to determine $\varDelta \varGamma _\mathrm {d}/\varGamma _\mathrm {d}$. The results are:

21 $$\begin{aligned} \varGamma _\mathrm {d}&= 0.662 \pm 0.003\,\text {(stat)} \pm 0.003\,\text {(syst)} \,\mathrm {ps}^{-1},\end{aligned}$$

22 $$\begin{aligned} \varDelta \varGamma _\mathrm {d}&= 0.023 \pm 0.015\,\text {(stat)} \pm 0.016\,\text {(syst)} \,\mathrm {ps}^{-1}, \end{aligned}$$

23 $$\begin{aligned} \varDelta \varGamma _\mathrm {d}/\varGamma _\mathrm {d}&= 0.034 \pm 0.023\,\text {(stat)} \pm 0.024\,\text {(syst)}. \end{aligned}$$

Neglecting CP violation in mixing, the measured $\mathrm {B}^0_\mathrm {s} \!\rightarrow \! \mathrm {J}/\psi \mathrm {\pi ^+}\mathrm {\pi ^-}$ lifetime can be translated into the width of the heavy $\mathrm {B}^0_\mathrm {s}$ mass eigenstate:

24 $$\begin{aligned} \varGamma _\mathrm {H} = 1/\tau _{\mathrm {B}^0_\mathrm {s}} = 0.596 \pm 0.012\,\text {(stat)} \pm 0.004\,\text {(syst)} \,\mathrm {ps}^{-1}. \end{aligned}$$

Solving for $c\tau _{\mathrm {L}}$ from Eq. (4) gives

25 $$\begin{aligned} c\tau _\mathrm {L} = \frac{1}{2} c\tau _{\text {eff}} + \sqrt{ \frac{1}{4} (c\tau _{\text {eff}})^2 - \frac{|A_\perp |^2}{1-|A_\perp |^2} c\tau _\mathrm {H}( c\tau _\mathrm {H} - c\tau _{\text {eff}})}. \end{aligned}$$

Using the $\mathrm {B}^0_\mathrm {s} \!\rightarrow \! \mathrm {J}/\psi \mathrm {\pi ^+}\mathrm {\pi ^-}$ result in Eq. (14), the measured $\mathrm {B}^0_\mathrm {s}$ effective lifetime in Eq. (15), and the world-average value of the magnitude squared of the CP-odd amplitude $|A_\perp |^2 = 0.250 \pm 0.006$ [2], the lifetime of the light component is found to be $c\tau _{\mathrm {L}} = 420.4 \pm 6.2\,\upmu \text {m}$. The uncertainty includes all statistical and systematic uncertainties, taking into account the correlated uncertainties. The result is consistent with the world-average value of $423.6 \pm 1.8\,\upmu \text {m}$ [5].

Our measured lifetimes for ${\mathrm {B}^0}$, $\mathrm {B}^0_\mathrm {s} \!\rightarrow \! \mathrm {J}/\psi \mathrm {\phi (1020)}$, and $\varLambda _\mathrm {b}^0$ are compatible with the current world-average values [5] of $455.7 \pm 1.2$, $443.4 \pm 3.6$, and $440.7 \pm 3.0$ $\,\upmu \text {m}$, respectively. In addition, our measurement of the $\mathrm {B}^0_\mathrm {s}$ lifetime in the $\mathrm {B}^0_\mathrm {s} \!\rightarrow \! \mathrm {J}/\psi \mathrm {\pi ^+}\mathrm {\pi ^-}$ channel is in agreement with the results from CDF, LHCb, and D0: $510\,^{+36}_{-33}\,\text {(stat)} \pm 9\,\text {(syst)}$ $\,\upmu \text {m}$  [30], $495.3 \pm 7.2\,\text {(stat)} \pm 7.2\,\text {(syst)}$ $\,\upmu \text {m}$  [31], and $508 \pm 42\,\text {(stat)} \pm 16\,\text {(syst)}$ $\,\upmu \text {m}$  [32], respectively.

Our final result for the $\mathrm {B}_\mathrm {c}^+$ lifetime using the $\mathrm {J}/\psi \mathrm {\pi ^+}$ mode is:

26 $$\begin{aligned} c\tau _{\mathrm {B}_\mathrm {c}^+} = 162.3 \pm 7.8\,\text {(stat)} \pm 4.2\,\text {(syst)} \pm 0.1 (\tau _{{\mathrm {B}^{+}}})\,\upmu \text {m},\nonumber \\ \end{aligned}$$

where the systematic uncertainty from the ${\mathrm {B}^{+}}$ lifetime uncertainty [5] is quoted separately in the result. This measurement is in agreement with the world-average value $(152.0 \pm 2.7\,\upmu \text {m})$ [5]. Precise measurements of the $\mathrm {B}_\mathrm {c}^+$ lifetime allow tests of various theoretical models, which predict values ranging from 90 to 210$\,\upmu \text {m}$  [33–36]. Furthermore, they provide new constraints on possible physics beyond the standard model from the observed anomalies in ${\mathrm {B}}\rightarrow \mathrm {D}^{(*)}\tau \nu$ decays [37].

## Summary

The lifetimes of the ${\mathrm {B}^0}$, $\mathrm {B}^0_\mathrm {s}$, $\varLambda _\mathrm {b}^0$, and $\mathrm {B}_\mathrm {c}^+$ hadrons have been measured using fully reconstructed decays with a $\mathrm {J}/\psi$ meson. The data were collected by the CMS detector in proton–proton collision events at a centre-of-mass energy of 8$\,\text {Te}\text {V}$, and correspond to an integrated luminosity of 19.7$\,\text {fb}^\text {-1}$. The ${\mathrm {B}^0}$ and $\mathrm {B}^0_\mathrm {s}$ meson lifetimes have each been measured in two channels: $\mathrm {J}/\psi \mathrm {K^{*}(892)}{}^{0}$, $\mathrm {J}/\psi \mathrm {K^0_S}$ for ${\mathrm {B}^0}$ and$\mathrm {J}/\psi \mathrm {\pi ^+}\mathrm {\pi ^-}$, $\mathrm {J}/\psi \mathrm {\phi (1020)}$ for $\mathrm {B}^0_\mathrm {s}$. The precision from each channel is as good as or better than previous measurements in the respective channel. The ${\mathrm {B}^0}$ lifetime results are used to obtain an average lifetime and to measure the decay width difference between the two mass eigenstates. The $\mathrm {B}^0_\mathrm {s}$ lifetime results are used to obtain the lifetimes of the heavy and light $\mathrm {B}^0_\mathrm {s}$ mass eigenstates. The precision of the $\varLambda _\mathrm {b}^0$ lifetime measurement is also as good as any previous measurement in the $\mathrm {J}/\psi \mathrm {\Lambda }^{0}$ channel. The measured $\mathrm {B}_\mathrm {c}^+$ meson lifetime is in agreement with the results from LHCb and significantly more precise than the CDF and D0 measurements. All measured lifetimes are compatible with the current world-average values.
